# Integrating multi-omics and deep learning to explore the active ingredients and molecular mechanisms of *Cinnamomum migao* volatile oil in the treatment of coronary heart disease

**DOI:** 10.3389/fphar.2026.1823482

**Published:** 2026-08-05

**Authors:** Xiaomei Zeng, Jiangtao Guo, Jian Xu, Yongping Zhang, Xiaoting Zhu, Jie Liu

**Affiliations:** 1 School of Pharmacy, Guizhou University of Traditional Chinese Medicine, Guiyang, China; 2 National Engineering Research Center of Miao’s Medicines, Guiyang, China; 3 AVIC 303 Hospital, Anshun, China

**Keywords:** *Cinnamomum migao* volatile oil, coronary heart disease, lipid metabolism, molecular docking, network analysis, serum pharmacochemistry, transcriptomics

## Abstract

**Background:**

Coronary heart disease (CHD) is a major cardiovascular disease. *Cinnamomum migao* volatile oil (CM-VO) is traditionally used for CHD, but its active basis and mechanisms remain unclear.

**Objective:**

To clarify the chemical basis and potential anti-CHD mechanisms of CM-VO.

**Methods:**

GC–MS was used to identify CM-VO constituents and serum-related metabolites. A mouse CHD model was established to evaluate pharmacodynamic effects. Network analysis was used for full-component mechanism prediction, while transcriptomics was used to screen treatment-responsive genes. UniProt standardization, deep learning, molecular docking, and H9c2 cell experiments were further performed for target prediction and validation.

**Results:**

A total of 68 CM-VO constituents and 8 serum-related metabolites were identified. CM-VO improved myocardial injury, inflammation, oxidative stress, endothelial dysfunction, and lipid-related abnormalities in CHD mice. Network analysis predicted 39 bioactive compounds and 58 key targets, while transcriptomics identified 44 DEGs and 101 CHD-related enriched genes. After UniProt standardization, 19 human genes were analyzed, and GADD45A, MTHFS, and ALAS2 were prioritized as high-affinity targets. In H9c2 cells, CM-VO-containing serum reduced lipid accumulation and injury markers, enhanced antioxidant capacity, and regulated GADD45A, MTHFS, and ALAS2 expression.

**Conclusion:**

CM-VO may protect against CHD mainly by regulating inflammation, oxidative stress, endothelial function, and lipid metabolism. This study provides integrated evidence for the pharmacological basis and molecular mechanisms of CM-VO against CHD.

## Graphical Abstract

**Figure:**
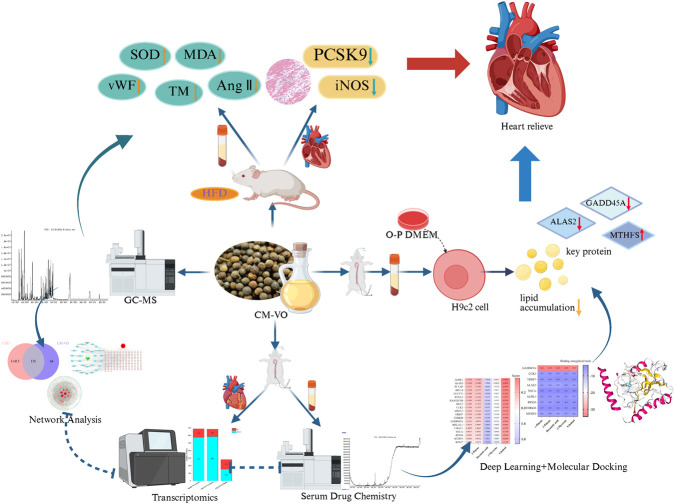

## 1 Introduction

Coronary heart disease (CHD) arises from atherosclerosis, characterized by localized abnormal thickening of the arterial intima. Its progression is driven by multiple factors including lipid metabolism disorders, vascular endothelial injury, inflammation, and immune dysfunction (Zhou et al., 2021). The pathological mechanisms involve complex, intertwined pathways related to lipid dysregulation, endothelial damage, inflammatory responses, and oxidative stress (Shaya et al., 2022; Guo et al., 2023; Dong et al., 2019). CHD ranks among the leading causes of death worldwide. According to World Health Organization statistics, cardiovascular diseases are responsible for approximately 17.9 million deaths annually, accounting for 31% of global mortality (World Health Organization WHO, 2021). In China, the incidence of CHD has risen significantly alongside population aging and lifestyle changes, making it a primary cause of death among both urban and rural residents (Zhao et al., 2019). Despite advances in modern treatments such as percutaneous coronary intervention (PCI) and pharmacological therapies, limitations remain, including high costs, side effects, and individual variations in efficacy (Benjamin et al., 2019). Traditional Chinese medicine (TCM), with its multi-component and multi-target characteristics, demonstrates therapeutic advantages in lipid regulation, anti-inflammation, and antioxidant effects (Wang et al., 2012). Several TCM preparations, such as Compound Danshen Droplet Pills (Yang et al., 2024), Tongmai Yangxin Pills (Chen et al., 2020), and Shexiang Tongxin Droplet Pills (Cui et al., 2023), are currently used clinically.

*Cinnamomum migao* H. W. Li (CM) refers to the dried mature fruit of *C. migao* H. W. Li, a medicinal plant belonging to the family Lauraceae (Li, 2010). Listed in the Guizhou Chinese and Ethnic Medicinal Materials Standards (2019 edition) (Guizhou Provincial Drug Administration, 2019), it is a traditional medicinal material used by the Miao ethnic group in Guizhou. It is characterized as pungent and warm in property, non-toxic, and possesses effects such as dispersing cold, eliminating dampness, promoting qi circulation, and relieving pain (Li et al., 2011). CM contains volatile oils, fatty oils, fatty acids, flavonoids, and inorganic elements (Huang et al., 2019). Research indicates that the volatile oil components constitute its primary active constituents (Min et al., 2021). The *C. migao* volatile oil (CM-VO) exhibits cardioprotective effects by alleviating myocardial ischaemia, relieving angina, stabilising electrocardiographic activity, and ameliorating myocardial structural damage through the inhibition of inflammation and oxidative stress. In isoproterenol-induced myocardial ischaemic rats, it reduces ST-segment elevation, improves ventricular repolarisation, and limits ischaemic injury (Jian et al., 2023), while enhancing left ventricular systolic pressure and maximal rates of pressure change, and lowering left ventricular end-diastolic pressure, thus improving both systolic and diastolic dysfunction (Yao et al., 2022). Clinically, Liqi Huoxue Diwuan and Migao Xinle Diguan, in which it is the principal ingredient, effectively treat stable exertional angina (cold-damp stagnation and blood stasis), relieving chest pain, tightness, palpitations and shortness of breath, and reducing nitroglycerin use (Yawen et al., 2005). The CM-VO downregulates miR-328, modulating L-type calcium channel-related proteins (CaV1.2, CaM, CaMKII, p-CaMKII) to reverse calcium overload and electrical remodelling, thereby ameliorating supraventricular arrhythmias and QT prolongation (Chanheng et al., 2024). It lowers serum cTn-I, CK-MB, LDH and AST, attenuates myocardial necrosis, inflammatory infiltration, and interstitial collagen deposition, inhibits fibrosis, and protects the aortic endothelial integrity (Yajun and Xin, 2020); the underlying mechanism involves enhancing superoxide dismutase activity, reducing malondialdehyde, and down-regulating IL-6 and TNF-α, thereby interrupting the inflammation–oxidative stress feedback loop. Its major constituents (1,8-cineole, cypressene, limonene, β-caryophyllene) act synergistically to dilate coronary arteries, increase coronary blood flow, slow heart rate, reduce myocardial oxygen consumption, and exert a mild antihypertensive effect, ultimately achieving the therapeutic outcome of increasing oxygen supply, reducing oxygen consumption, protecting the myocardium, and regulating cardiac rhythm (Yawen et al., 2005). However, the precise chemical composition of CM-VO and its pharmacodynamic mechanisms against CHD remain incompletely elucidated, limiting its further development and application.

Gas chromatography (GC) separates complex mixtures based on differences in distribution coefficients between stationary and mobile phases. Mass spectrometry (MS) enables qualitative and quantitative analysis by detecting the mass-to-charge ratios of ion fragments. The coupling of GC and MS (GC-MS) allows for effective analysis of volatile and semi-volatile components (Rodríguez-Maec et al., 2017). Serum pharmacochemistry identifies drug-derived components and metabolites transferred into the bloodstream after administration, serving as a bridge between medicinal chemistry and pharmacology (Ma et al., 2017). Network analysis (NA), a computational approach widely applied in ethnopharmacology, operates within a systems biology framework to systematically interrogate complex interaction networks involving natural products, chemical compounds, therapeutic targets, and signaling pathways. This methodology facilitates the prediction of potential targets and generates testable hypotheses regarding the multi-target mechanisms of action underlying natural product-based therapies (Diao et al., 2026). Transcriptomics employs high-throughput sequencing to profile genome-wide gene expression changes, elucidating key pathways and biological processes regulated by drugs (Wang et al., 2009). Deep learning processes high-dimensional omics data through multi-layer neural networks, offering advantages such as automatic feature extraction (Yu et al., 2022). Molecular docking uses computational simulations to predict, at the atomic level, the binding patterns and affinities between small molecules and target proteins, providing structural insights into interactions (Meng et al., 2011). Drug-containing serum, prepared via oral administration to animals, is used in cellular experiments to study drug effects under near-physiological conditions, circumventing issues related to direct *in vitro* drug toxicity and concentration distortion. Although these analytical methods differ in their technical principles, they all serve the core objective of elucidating the pharmacological basis and mechanisms of action, together forming a progressive research chain from chemical characterization to functional validation. Moreover, they consistently adopt a multi-target, systems-based perspective, rejecting the reductionist mindset of single-target approaches. Their core differences lie in the analytical targets, scope of coverage and validation logic. In terms of analytical targets, GC-MS and serum pharmacochemistry focus on chemical composition, whereas transcriptomics and drug-containing serum experiments address biological effects. Regarding scope, targeted analysis and genome-wide screening complement each other, with network analysis integrating the data. In terms of validation logic, a cyclical, iterative process is established through the generation and verification of hypotheses. It should be noted that serum pharmacochemistry, which differs in nature from drug-containing serum, provides mutual corroboration. In summary, with their clear division of labor and seamless integration, these technologies collectively constitute a comprehensive, integrated research network.

This study aimed to systematically investigate the material basis and mechanisms of CM-VO in treating CHD using a multidisciplinary approach. First, GC-MS was used to analyze the chemical composition of CM-VO and its bioavailable components in serum after administration. Second, a mice CHD model was established to evaluate the effects of CM-VO on cardiac function, blood lipids, and myocardial histopathology. Next, an integrated strategy combining bioinformatics screening of bioactive compounds and their targets with transcriptomic analysis of differentially expressed genes enabled the construction of a network map for the identification of key compounds and core targets. Subsequently, deep learning combined with molecular docking was used to assess the binding affinity between bioavailable components and transcriptional targets. Palmitic acid (PA) is the most abundant saturated long-chain fatty acid in human plasma, whilst oleic acid (OA) is the primary monounsaturated fatty acid. The levels of both fatty acids are elevated in the plasma of individuals at high risk of coronary heart disease and are closely associated with the onset and progression of atherosclerosis (AS). Palmitic acid can induce vascular endothelial damage, inflammatory activation and cellular lipid deposition, whilst oleic acid promotes the proliferation of vascular smooth muscle cells; the combined action of these two fatty acids synergistically activates key atherosclerotic signalling pathways such as NF-κB and mTOR, upregulates the expression of adhesion molecules and vascular endothelial growth factor (VEGF), and induces lipid deposition, foam cell transformation, and abnormal proliferation and migration of smooth muscle cells, thereby accurately simulating the early pathological features of atherosclerosis; This modelling approach is stable, controllable and reproducible, and is consistent with the pathogenesis of atherosclerotic (AS) cells reported in the literature (Arai, 2015; He et al., 2025; Zhang et al., 2024; Lamers et al., 2011). Therefore, in this study, the predicted targets were validated using CM-VO-containing serum in a palmitic acid/oleic acid-induced H9c2 cardiomyocyte model. This work provides a scientific basis for the advanced development of CM-VO and novel therapeutic strategies for CHD.

## 2 Materials and methods

### 2.1 Experimental reagents and materials

*Cinnamomum migao* (collected from Luodian Town, Luodian County, Guizhou Province) were identified by Professor Sun Qingwen (Guizhou University of Chinese Medicine) as the dried mature fruit of *C. migao* H. W. Li. Atorvastatin calcium tablets (Lupu Pharmaceutical Technology Co., Ltd., batch no. 202211120B) and nicorandil tablets (Xi’an Hanfeng Pharmaceutical Co., Ltd., batch no. 2302051) were used as reference drugs. Other reagents and materials are listed in Supplementary Appendix A.

### 2.2 Principal instrumentation

Agilent gas chromatography-single quadrupole mass spectrometry system (USA, 19091S-436); Electronic balance (Mettler Toledo Instruments (Shanghai) Co., Ltd., ME203E/02); High-speed benchtop refrigerated centrifuge (Eppendorf 5424R); Laminar flow hood (Suzhou Antai Air Co., Ltd., SW-CJ-2F). Other experimental instruments are detailed in Supplementary Appendix B.

### 2.3 Experimental animals and cells

ICR mice (male and female, 18–22 g) were purchased from Hangzhou Ziyuan Laboratory Animal Technology Co., Ltd., (license no. SCXK (Zhe) 2019-004). Sprague Dawley (SD) mice (SPF grade, male and female, 250–300 g) were obtained from Changsha Tianqin Biological Co., Ltd., (production certificate: SCXK (Xiang) 2019-0014). All animals were quarantined before use. Animal experimental protocols were approved by the Laboratory Animal Ethics Committee of Guizhou University of Chinese Medicine (approval no. 20250721005).

The H9c2 cardiomyocyte cell line was provided by Shang’en Biotechnology Co., Ltd.

### 2.4 Methods

#### 2.4.1 Chemical composition analysis of CM-VO

##### 2.4.1.1 Extraction of CM-VO

Precisely 100 g of coarse powdered CM was subjected to steam distillation under optimized conditions (material-to-liquid ratio 1:5 g/mL, soaking time 5 h, extraction time 8 h) (Jie et al., 2022). Following distillation, the collected oil phase was centrifuged at 13,000 rpm for 10 min. The upper clear liquid layer was carefully aspirated to obtain CM-VO.

##### 2.4.1.2 GC-MS analysis

Analysis was performed using an Agilent 19091S-436 GC-MS system equipped with an HP-5MS column (60 m × 250 μm × 0.25 μm). Chromatographic conditions: initial temperature 50 °C held for 2 min, increased to 200 °C at 6 °C/min, then to 310 °C at 10 °C/min and held for 20 min. High-purity nitrogen was the carrier gas; injection volume 1 μL. MS conditions: quadrupole temperature 150 °C, full scan mode (m/z 29-500), solvent delay 8.00 min, ion source temperature 230 °C. Data were acquired and processed using Agilent workstation software. Constituents were preliminarily identified by matching against the NIST 2020 standard library.

#### 2.4.2 Analysis of CM-VO components entering the bloodstream

##### 2.4.2.1 Preparation of CM-VO-containing serum

CM-VO was suspended in 0.5% CMC-Na solution. SD rats were divided into blank and CM-VO groups (n = 6 per group), and the CM-VO group was orally administered 123.48 mg/kg CM-VO for 7 days. Blood samples were collected from the orbital sinus at 0, 15, and 30 min, and 1, 1.5, 2, 3, 4.5, 6, and 8 h after administration. After standing for 30 min, samples were centrifuged at 3,000 rpm for 15 min to obtain serum. Serum samples from different time points were pooled and mixed. Then, 200 μL serum was precipitated with four volumes of methanol, vortexed for 30 s, and centrifuged at 12,000 r/min for 10 min at 4 °C. The supernatant was dried under nitrogen at 40 °C, reconstituted in 50% methanol, centrifuged again, and subjected to instrumental analysis. Blank serum was processed in the same manner.

##### 2.4.2.2 GC-MS detection and analysis

Analysis was performed as described in Section 2.4.1.2. Mass spectrometry data acquired from drug-containing serum and blank serum samples prepared via CM-VO were processed using MSD software. Compounds were tentatively identified by matching against the NIST 2020 mass spectral library. Endogenous serum components were subtracted, and the resulting spectra were compared with those obtained from CM-VO *in vitro* extracts to obtain preliminary identifications. Subsequently, the secondary fragment ions were analyzed in greater detail, and by integrating these fragmentation data with relevant literature, the *in vivo* metabolic pathways and cleavage patterns were inferred.

#### 2.4.3 Pharmacodynamic study of CM-VO in a mice CHD model

##### 2.4.3.1 Model establishment

Cold water immersion combined with a high-fat diet (HFD) is a well-established mice model for atherosclerosis-related coronary heart disease. Cold stress accelerates plaque growth and vulnerability by activating UCP1-dependent lipolysis, elevating atherogenic lipids, and promoting vascular inflammation and oxidative stress, while HFD induces persistent hyperlipidemia. Together, this combined protocol reliably recapitulates the dyslipidemia, endothelial injury, inflammation, and plaque instability characteristic of human coronary heart disease (Dong et al., 2013; Yang et al., 2025; Yunling et al., 2022). Mice were subjected to cold water immersion (0 °C–2 °C) for 20 min daily for two consecutive weeks, concurrently fed a high-fat diet (1% cholesterol, 10% lard, 0.2% propylthiouracil, 0.5% bile salts, 5% egg yolk powder, 5% sucrose, 78.3% basal diet) for 3 weeks (Dingfa et al., 2022). Electrocardiogram (ECG) in lead II was monitored using a BL-420N system. Model success was assessed based on general behavior, body temperature, body weight, ECG, and hematoxylin and eosin (HE) staining results.

##### 2.4.3.2 Grouping and administration

Successfully modeled mice were randomly assigned to six groups (n = 12 per group): model, atorvastatin (AC, 2.6 mg/kg), nicorandil (N, 1.3 mg/kg), low-dose CM-VO (CM-VOL, 13.72 mg/kg), medium-dose CM-VO (CM-VOM, 41.16 mg/kg), and high-dose CM-VO (CM-VOH, 123.48 mg/kg). An additional group of 12 unmodeled mice served as the blank control (control). Control and model groups received equal volumes of saline. CM-VO doses were prepared in 0.5% CMC-Na solution based on clinical dosing standards (The dosage of CM-VO is calculated based on the clinical dosage of Liqi Huoxue Diwan. The recommended daily dose of Liqi Huoxue Diwan for adults is 0.75 g; calculations show that this requires 3.375 g of raw Alpinia officinarum, with a VO yield of 9.38%). Administration continued for 4 weeks. General condition, mortality, and body weight were recorded.

##### 2.4.3.3 ECG monitoring in lead II

Mice were anesthetized with 0.3% sodium pentobarbital, positioned supine, and connected to the BL-420N system via subcutaneous needle electrodes in the limbs. ECG changes were recorded after stabilization.

##### 2.4.3.4 Tissue harvesting and Sample preparation

After treatment, all mice were fasted for 12 h with free access to water. In each group, a subset of mice was used for blood collection from the abdominal aorta. The blood was allowed to stand at room temperature for 30 min and then centrifuged at 3,500 r/min for 15 min at 4 °C. The supernatant serum was harvested and stored at −80 °C for subsequent analyses. The hearts from these mice were rapidly dissected and fixed in 4% paraformaldehyde solution for later hematoxylin and eosin (HE) staining. The remaining mice in each group were anesthetized by intraperitoneal injection of 0.3% sodium pentobarbital and transcardially perfused with saline to remove blood. Heart tissues were then collected for Western blot analysis.

##### 2.4.3.5 Cardiac tissue histopathology

After fixation, the mice hearts were removed from 4% paraformaldehyde and rehydrated under running tap water for 12 h. The cardiac tissue was then dehydrated, embedded in paraffin and demoulded, followed by demoulding into water. Following sectioning, the tissue sections were treated with High-Definition Constant Staining Pretreatment Solution for 1 min, stained with hematoxylin for 5 min, and rinsed with tap water. The sections were briefly differentiated, rinsed again with tap water, counterstained with counterstain solution, and finally rinsed with tap water. Subsequently, the sections were dehydrated by sequential immersion in 85% ethanol and 95% ethanol for 5 min each, followed by staining with eosin solution for 5 min. The sections were then subjected to a series of dehydration and clearing steps: anhydrous ethanol I, II, and III for 5 min each, followed by eco-friendly dewaxing solution I and II for 5 min each. Finally, the sections were mounted with neutral resin. The stained sections were examined under a microscope, and images were captured and analyzed.

##### 2.4.3.6 ELISA assay

Serum levels of E-selectin, SOD, MDA, TM, vWF, EDRF, and Ang II were determined using specific ELISA kits according to the manufacturers' instructions.

##### 2.4.3.7 Western blotting assay

Mice heart tissue stored at −80 °C was minced into small pieces and lysed in 10 volumes of RIPA buffer containing phosphatase and protease inhibitors (100:1:1). The tissue was homogenized on ice and incubated on ice for 30 min with shaking every 10 min. The homogenate was transferred to 1.5 mL Eppendorf tubes and centrifuged at 12,000 rpm for 15 min at 4 °C, and the supernatant was collected. Protein samples were diluted with PBS at a 19:1 ratio. BSA standards (0.2–1.0 mg/mL) were prepared. Diluted samples (three replicates), standards (two replicates), and PBS (two replicates) were dispensed into a 96-well plate. BCA working solution (A:B = 50:1) was added (200 μL/well), and the plate was incubated at 37 °C for 30 min in the dark. Absorbance was measured at 562 nm using a microplate reader, and protein concentrations were calculated from the standard curve. Protein concentrations were normalized with RIPA buffer, mixed with 5× loading buffer at a 4:1 ratio, denatured in a metal bath for 5 min, cooled, and stored at −20 °C. Following SDS-PAGE gel preparation, 30 μg of protein per lane and a molecular weight marker were loaded. Electrophoresis was performed at 80 V until the bromophenol blue reached the separating gel, then at 120 V until it reached the bottom. For protein transfer, the NC membrane, filter paper, and gel were soaked in transfer buffer and assembled in the order: black plate–sponge–filter paper–gel–NC membrane–filter paper–sponge–white plate. Transfer was conducted at a constant current of 200 mA for 2 h. The membrane was blocked with 5% skim milk in TBST for 2 h at room temperature, then incubated overnight at 4 °C with primary antibodies: GAPDH (1:2,000), PCSK9 (1:2,000), and iNOS (1:1,000). After three 10-min washes with TBST, the membrane was incubated with secondary antibody (1:5,000) for 2 h at room temperature, followed by three additional TBST washes. ECL reagent was applied dropwise, and protein bands were visualized using a gel imaging system. Grey-scale values were analyzed using ImageJ.

#### 2.4.4 Network analysis-based mechanism prediction and transcriptomic target screening

##### 2.4.4.1 Screening of CM-VO active components and targets

The 68 identified CM-VO constituents were screened for activity using TCMSP (drug-likeness ≥ 0.18, oral bioavailability ≥ 30%) and SwissADME (meeting ≥ 2 pharmacophore rules, gastrointestinal absorption “High”) databases. Bioactive components were input into SwissTargetPrediction (probability > 0) and TCMSP to predict target genes (Huan et al., 2024). Gene symbols were standardized via UniProt.

##### 2.4.4.2 CHD target prediction

CHD-related genes were retrieved from Genecards, OMIM, PharmGKB, TTD, and DrugBank databases using keywords “Coronary Artery Disease” and “Coronary Heart Disease” (Dongping et al., 2024). Genes with a Genecards Relevance score ≥1 and OMIM-listed CHD genes were selected. Targets from all databases were consolidated.

##### 2.4.4.3 Screening of core therapeutic targets

The intersection of CM-VO component targets and CHD targets was identified using the MicroBioinformatics platform. These intersecting targets were imported into the STRING database (species: *Homo sapiens*, combined score >0.4) to construct a protein-protein interaction (PPI) network, which was visualized and analyzed using Cytoscape 3.8.2. Topological parameters (degree, betweenness centrality, closeness centrality) were calculated, and nodes with median values were designated as key targets (Yujie et al., 2023).

##### 2.4.4.4 Transcriptomics analysis

After successful model establishment, mice were randomly divided into three groups (n = 3 per group): a control group (untreated), a model group, and an CM-VO treatment group. Mice in the CM-VO group received a daily dose of 123.5 mg/kg body weight of CM-VO by oral gavage, while the control and model groups were administered an equal volume of 0.9% saline solution. All treatments were administered continuously for 4 weeks. At the end of the treatment period, mice were anesthetized with sodium pentobarbital and perfused transcardially with saline; cardiac tissues were then collected, immediately frozen in liquid nitrogen, and stored at −80 °C until further analysis. Total RNA was extracted using TRIzol reagent (Invitrogen, United States) in combination with a purification kit according to the manufacturer’s instructions. RNA concentration and purity were assessed using a NanoDrop 2000 spectrophotometer (Thermo Fisher Scientific, United States), and integrity was evaluated by agarose gel electrophoresis and further confirmed with an Agilent 5300 Bioanalyzer (Agilent Technologies, United States). Only samples meeting the following quality criteria were used for subsequent library construction: total RNA ≥ 1 μg, concentration ≥ 30 ng/μL, RNA integrity number (RIN) > 6.5, and OD260/280 ratio between 1.8 and 2.2. Sequencing libraries were constructed following the Illumina NovaSeq Reagent Kit protocol, which involved mRNA isolation and purification, reverse transcription, 3′-end modification, adapter ligation, and PCR amplification. The resulting libraries were sequenced on the Illumina NovaSeq Xplus platform using paired-end sequencing. For bioinformatics analysis, raw sequencing reads were processed using fastp to obtain clean reads by removing low-quality data and adapter sequences. The clean reads were aligned to the reference genome using HiSat2, and gene expression levels were quantified with RSEM. Differential expression analysis was performed using the DESeq2 package, with genes meeting the criteria of FDR < 0.05 and |log2FC| ≥ 1 considered significantly differentially expressed.

##### 2.4.4.5 UniProt-based human standardization of DEGs

To ensure consistency with human target databases, mouse-derived differentially expressed genes (DEGs) were standardized using UniProt before downstream analysis. DEGs symbols were submitted to UniProt with the organism restricted to “*Homo sapiens*”. Matched human gene, including gene symbols, UniProt accession numbers, were retained, whereas genes lacking reliable human annotations were excluded. The resulting human-standardized gene set was used for subsequent network analysis, deep learning prediction, and molecular docking.

##### 2.4.4.6 GO and KEGG enrichment analysis

DEGs and key network analysis targets were pooled and analyzed using the Metascape database (P < 0.01) for Gene Ontology (GO) functional annotation and Kyoto Encyclopedia of Genes and Genomes (KEGG) pathway enrichment. These enriched terms were visualized via the MicroBioinformatics platform.

#### 2.4.5 Deep learning prediction of protein-ligand binding affinity

##### 2.4.5.1 Deep learning model

SMILES strings were retrieved from PubChem, and protein structures were obtained from the RCSB PDB. Amino acid sequences were cross-validated using SwissTargetPrediction (*H. sapiens*). A compound–protein dataset was constructed from all valid PubChem–PDB/SwissTargetPrediction matches, with sample size determined by available pair combinations. Compounds were encoded using Morgan fingerprints (radius = 2, 1024 bits), and proteins were embedded using ProtBERT with tokenisation, contextual encoding, and average pooling. The two feature vectors were concatenated for binding affinity prediction within a regression framework. The dataset was split into training and test sets (8:2), and five-fold cross-validation was applied on the training set to reduce overfitting and improve robustness. Four regression models (linear regression, random forest, SVR, and gradient boosting) were evaluated. The gradient boosting model performed best and was selected. Its hyperparameters were set as: learning rate = 0.01, n_estimators = 100, max_depth = 8, min_samples_split = 2, and min_samples_leaf = 1, optimized via cross-validatioModel performance was assessed using MSE, RMSE, MAE, and R^2^. The final model was used to predict compound–target binding affinities, and interaction pairs were ranked by predicted scores.

##### 2.4.5.2 Molecular docking

Molecular docking was employed to elucidate protein–ligand interactions at the molecular level and to evaluate the binding affinity and stability of the docked complexes. CB-Dock2, a blind docking online tool released in 2022 by Sichuan University, was utilized for this purpose. This server integrates structure-based cavity detection with homology-based template matching, leveraging the BioLiP template library and the FitDock algorithm to simultaneously predict binding pockets and ligand binding conformations without prior knowledge of binding sites, thereby enhancing docking accuracy. For the docking analysis, three-dimensional crystal structures of the target proteins were obtained from the RCSB Protein Data Bank (PDB; https://www.rcsb.org/). The three-dimensional structures of the bioactive compounds were downloaded from the PubChem database (https://pubchem.ncbi.nlm.nih.gov/). These small molecules, serving as ligands, were imported into CB-Dock2 (https://cadd.labshare.cn/cb-dock2/php/blinddock.php) for subsequent blind docking.

#### 2.4.6 Protective effects against lipid metabolism abnormalities in H9c2 cardiomyocytes

##### 2.4.6.1 Animal grouping and drug administration

SD rats (n = 20 per group) were divided into normal, nicorandil (positive control, 3 mg/kg), and CM-VO (85.47 mg/kg) groups. Drugs or vehicle (0.5% CMC-Na) were administered via gavage (10 mL/kg) daily for 7 days. Blood was collected at multiple time points post-final administration. Serum was pooled within groups, heat-inactivated (56 °C, 30 min), filtered (0.22 μm), aliquoted, and stored at −80 °C.

##### 2.4.6.2 Preparation of drug-containing serum

Both blank serum and drug-containing serum were diluted to the corresponding volume fractions with DMEM medium prior to use.

##### 2.4.6.3 Culture of H9c2 cardiomyocytes

Cells were thawed following the “slow freezing, rapid thawing” principle: cryovials retrieved from liquid nitrogen were immediately immersed in a 37 °C water bath with gentle agitation. The cell suspension was rapidly transferred to a centrifuge tube containing pre-warmed complete medium and centrifuged at 1,000 rpm for 5 min. The supernatant was discarded, and the cell pellet was resuspended in fresh complete medium by gentle pipetting, then seeded into culture flasks and maintained at 37 °C in a 5% CO_2_ incubator. At 48 h post-thaw, adherent cells were examined under an inverted microscope, washed twice with phosphate-buffered saline (PBS), and replenished with fresh complete medium before further incubation. Upon reaching approximately 80% confluence, cells were passaged: the culture medium was removed, and the monolayer was rinsed twice with PBS, followed by digestion with 0.25% trypsin at 37 °C for 30–60 s until cells became rounded and detached. Digestion was terminated by adding three volumes of complete medium, and the cell suspension was centrifuged at 1,000 rpm for 5 min. The supernatant was discarded, and the cell pellet was resuspended in 3 mL of complete medium. Cells were subsequently subcultured at a 1:2 ratio and maintained under the same conditions. For cryopreservation, cells were harvested following the same washing, digestion, and centrifugation procedures. The cell pellet was resuspended in cryopreservation medium consisting of DMEM, fetal bovine serum (FBS), and dimethyl sulfoxide (DMSO) at a ratio of 7:2:1. Aliquots of 1 mL were transferred to appropriately labeled cryovials, placed in a controlled-rate freezing container, and stored at −80 °C.

##### 2.4.6.4 Determination of optimal serum concentration

H9c2 cells in the logarithmic growth phase were harvested and digested with 0.25% trypsin for 1 min. The cells were resuspended in complete culture medium at a density of 1 × 10^5^ cells/mL, and 100 μL of the suspension was seeded into each well of a 96-well plate. Each experimental group contained six replicate wells, and the peripheral wells were filled with sterile PBS to minimize edge effects. The plates were incubated for 24 h to allow cell attachment. Following attachment, the culture medium was removed, and the cells were treated with varying concentrations of blank serum (40%, 30%, 20%, 10%, 5%, 2.5%, and 0%) for 24 h, with the exception of the control group, which received no treatment. Subsequently, 100 μL of 10% CCK-8 reagent was added to each well, and the plates were incubated for an additional 2 h. Absorbance was measured at 450 nm using a microplate reader. Cell viability was calculated according to the formula: $\text{Cell survival rate}=\left(A_{\text{experiment}}-A_{\text{blank}}\right)/\left(A_{\text{control}}-A_{\text{blank}}\right)$ . The optimal concentration of drug-containing serum was determined based on the viability results. For experiments involving drug-containing serum from the positive drug and CM-VO, the same screening procedure was applied. Based on the blank serum results, six experimental groups were established: a control group (high-glucose medium supplemented with 20% blank serum), groups treated with 20%, 10%, 5%, and 2.5% drug-containing serum (each adjusted to the final volume with blank serum), and a 0% drug-containing serum group (high-glucose medium only). The experimental procedure was identical to that used for blank serum treatment. This approach enabled the identification of the optimal addition concentrations for both types of drug-containing sera.

##### 2.4.6.5 Establishment of a lipid metabolism abnormality model

Palmitic acid (PA; 0.056 g) and oleic acid (OA; 0.061 g) were dissolved in 0.1 mol/L NaOH and saponified in a 75 °C water bath for 2 h to obtain a 50 mmol/L solution of sodium palmitate and sodium oleate. The sodium oleate solution was then conjugated with 10% fatty acid-free bovine serum albumin (BSA) to prepare a 10 mmol/L stock solution, while the sodium palmitate solution was conjugated with 20% BSA to prepare a 20 mmol/L stock solution. Both stock solutions were sterilized by filtration through a 0.22 μm filter and stored at −4 °C until use. Of note, PA alone was not used in subsequent experiments due to its significant tissue damage, which renders it unsuitable for lipid-regulating screening. To assess the effect of O-P on cell viability, the stock solutions were diluted to five concentration gradients ranging from 0.16–0.08 to 2.5–1.25 mmol/L. H9C2 cells were seeded into 96-well plates at a density of 1 × 10^5^ cells/mL (100 μL per well). After 24 h of incubation, the plates were divided into seven groups: blank control, normal control, and five O-P treatment groups at different concentrations, each with six replicates. Following 24 h of drug exposure, cell viability was assessed by measuring absorbance at 450 nm using a microplate reader. For lipid accumulation analysis, H9C2 cells were seeded into 6-well plates at a density of 3 × 10^5^ cells/mL (1 mL per well). After cell attachment, the medium was replaced with high-glucose DMEM containing the indicated concentrations of O-P, and cells were cultured for an additional 24 h. The medium was then discarded, and cells were washed twice with PBS, fixed with 4% paraformaldehyde for 10 min, and washed twice again with PBS. Cells were dehydrated in 60% isopropanol for 10 min and washed twice with PBS. Freshly prepared Oil Red O staining solution (preheated at 37 °C for 30 min, saturated solution:water = 3:2) was added (1 mL/well), and cells were stained at 37 °C for 30 min. After staining, cells were washed twice with PBS, followed by a 30 s wash with 60% isopropanol. Images were captured under a microscope for further analysis.

##### 2.4.6.6 Intervention with CM-VO-containing serum

This experiment was divided into 6 groups. The control group: 100 μL of cell culture medium was added to each well, and the cells were cultured for 24 h; the model group: O-P concentration was 1.25–0.63 mmol·L^−1^, 100 μL per well, stimulation time was 12 h; the positive group: contained low-dose, medium-dose and high-dose CM-VO serum groups: first, the serum containing the drug was incubated for 12, 24, and 48 h respectively, then the O-P concentration of 1.25–0.63 mmol·L^−1^ was used for stimulation for 12 h.

##### 2.4.6.7 Measurement of biochemical indicators

Intracellular lipid levels (TG, CHO, LDL), oxidative stress markers (GSH, SOD, MDA), and myocardial injury markers (cTnT by ELISA; AST, LDH by automated analyzer) were measured.

##### 2.4.6.8 qRT-PCR

RNA Extraction, following drug treatment in 6-well plates, the culture medium was removed, and the cells were washed once with 1 mL of ice-cold phosphate-buffered saline (PBS). Subsequently, 1 mL of RNA extraction reagent was added to each well to lyse the cells. After 5 min of incubation at room temperature, the lysates were collected into sterile centrifuge tubes. A chloroform substitute (100 μL) was added to each tube, thoroughly mixed, and incubated on ice for 3 min. The samples were then centrifuged at 12,000 rpm for 10 min at 4 °C. An aliquot of 400 μL of the upper aqueous phase was carefully transferred to a new tube and mixed with 550 μL of isopropanol. After incubation at −20 °C for 15 min, the mixture was centrifuged at 12,000 rpm for 10 min at 4 °C. The supernatant was discarded, and the RNA pellet was washed 10–11 times with 1 mL of 75% ethanol, with centrifugation at 12,000 rpm for 5 min at 4 °C after each wash. The pellet was air-dried and dissolved in 15 μL of RNase-free water. RNA concentration and purity were assessed using a NanoDrop 2000 spectrophotometer. Primer Sequences, the primers (Table 1) were synthesized for quantitative real-time PCR (qPCR). Reverse Transcription, total RNA was reverse transcribed using the SweScript All-in-One First-Strand cDNA Synthesis Kit according to the manufacturer’s instructions. The reaction mixture (20 μL total volume) contained 4 μL of 5× SweScript All-in-One SuperMix, 1 μL of gDNA Remover, 10 μL of total RNA (1 μg), and RNase-free water to adjust the volume. The thermal cycling conditions were as follows: 25 °C for 5 min, 42 °C for 30 min, and 85 °C for 5 s. Quantitative Real-Time PCR (qPCR), qPCR was performed using the Universal Blue SYBR Green qPCR Master Mix on a real-time PCR detection system. Each reaction (15 μL total volume) consisted of 7.5 μL of 2× Master Mix, 1.5 μL of primer mix (2.5 μM each of forward and reverse primers), 2.0 μL of cDNA template, and 4.0 μL of RNase-free water. The amplification protocol was as follows: initial denaturation at 95 °C for 30 s, followed by 40 cycles of denaturation at 95 °C for 15 s and annealing/extension at 60 °C for 30 s. Melting curve analysis was performed by heating from 65 °C to 95 °C with fluorescence readings taken at 0.5 °C intervals. Relative gene expression levels were calculated using the 2^(−ΔΔCT)^ method. Briefly, the ΔCT value for each sample was obtained by subtracting the CT value of the internal control gene (GAPDH) from that of the target gene. The ΔΔCT was then calculated as the difference between the ΔCT of the treated sample and the ΔCT of the control sample. The fold change in gene expression was determined as 2^(−ΔΔCT)^.

**TABLE 1:** List of primer sequences.

Gene	Forward primer (5′→3′)	Reverse primer (5′→3′)	Product size (bp)
Rat-GADD45A	GCA​GAG​CAG​AAG​ATC​GAA​AGG	GCA​CGA​ATG​AGG​GTG​AAA​TGG	215
Rat-MTHFS	GCA​AGA​TGA​AAT​TGA​GAC​GGA​A	CAG​GAA​GGA​AGA​TGA​GGT​CAA​GTC	233
Rat-ALAS2	CAG​GCT​TCA​TCT​TTA​CCA​CTT​CAC​T	ATT​AGT​AGC​TGG​CGC​ATA​TGT​TTG​A	142
Rat-GAPDH	CTG​GAG​AAA​CCT​GCC​AAG​TAT​G	GGT​GGA​AGA​ATG​GGA​GTT​GCT	138

##### 2.4.6.9 Western blot

Following drug treatment, cells were washed twice with ice-cold PBS and lysed in 50 μL/well RIPA buffer containing PMSF (100:1). Lysates were collected by scraping, incubated on ice for 30 min with vortexing every 5 min, and centrifuged at 12,000 rpm for 5 min at 4 °C. Protein concentrations in the supernatants were determined by BCA assay using BSA standards (0–1,000 μg/mL), with absorbance measured at 562 nm. Samples were normalized with lysis buffer, mixed with 5× SDS loading buffer, and heated at 95 °C for 10 min. For Western blotting, proteins were separated by SDS-PAGE (pre-run at 100 V for 20 min, then 120 V), transferred to methanol-activated PVDF membranes (semi-dry, 200 mA, 90 min), and blocked with 5% skim milk in TBST for 1 h. Membranes were incubated overnight at 4 °C with primary antibodies (GAPDH 1:2,000; GADD45A, ALAS2, MTHFS 1:1,000), washed with TBST, then incubated with HRP-conjugated secondary antibody (1:10,000) for 1 h. Bands were visualized using ECL substrate and imaged; densitometric analysis was performed with ImageJ, normalizing target protein levels to GAPDH.

#### 2.4.7 Statistical analysis

Data are presented as mean ± standard deviation. Normality and homogeneity of variance were tested using SPSS 26.0. One-way analysis of variance (ANOVA) was used for intergroup comparisons. P < 0.05 was considered statistically significant. Graphs were generated using GraphPad Prism 8.0.2.

## 3 Results

### 3.1 Chemical composition analysis of CM-VO

**FIGURE 1:**
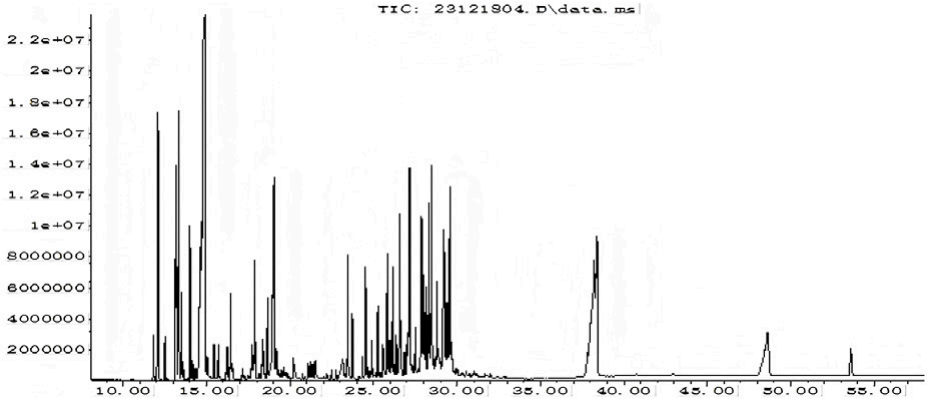
GC-MS chromatograms of CM-VO samples.

Mass spectrometry data collected from CM-VO were processed using MSD software. Compounds were identified using the NIST 2020 standard library. A total of 68 identified chemical constituents were detected, comprising 36 monoterpenes, 24 sesquiterpenes, 1 fatty acid, 3 fatty acid esters, 2 ketones, and one each of an aromatic alcohol and a quinone. The total ion current (TIC) profile and chemical composition are presented in Figure 1 and Supplementary Appendix C.

### 3.2 Analysis of CM-VO components in blood

**FIGURE 2:**
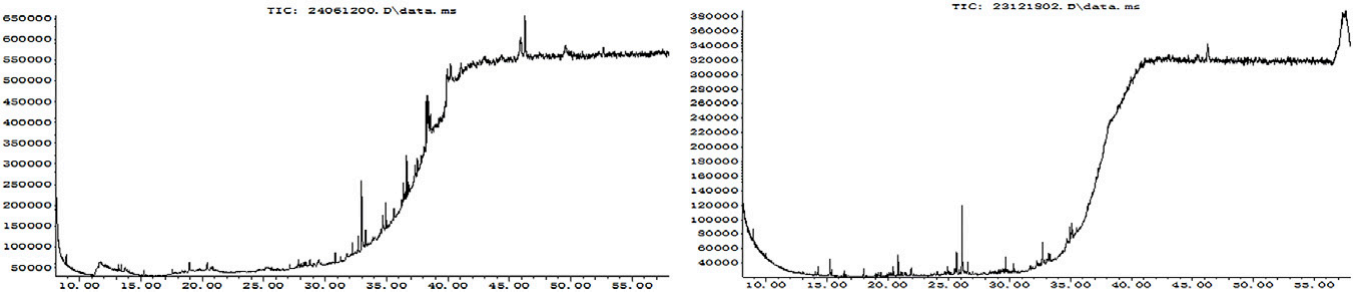
Total ion current chromatogram of serum (Left Blank serum; Right total ion current diagram of CM-VO).

Analysis of drug-containing serum, after subtracting endogenous peaks and comparing with *in vitro* CM-VO profiles (Figure 2), preliminarily identified 8 CM-VO-related metabolites (Table 2).

**TABLE 2:** Drug-containing serum and metabolites.

Peak	Chemical name	Molecular formula	MW	Exact Mass	R.T.	Area
M1	9-Octadecen-12-ynoic acid, methylester	C_19_H_32_O_2_	292	292.2402	8.94	349588
M2	Dodecane, 5,8-diethyl-	C_16_H_34_	226	226.2660	15.24	483291
M3	Tetradecane, 2,6,10-trimethyl-	C_17_H_36_	240	240.2817	20.85	645215
M4	Heptadecane, 9-hexyl-	C_23_H_48_	324	324.3756	21.91	302529
M5	Heptadecane, 2,6,10,15-tetramethyl-	C_21_H_44_	296	296.3443	25.64	815314
M6	Tetratetracontane	C_44_H_90_	618	618.7042	26.55	497815
M7	Stearic acid, 3-(octadecyloxy)propylester	C_39_H_78_O_3_	594	594.5950	31.7	136,744
M8	1,2-Propanediol, 3-(octadecyloxy)-, diacetate	C_25_H_48_O_5_	428	428.3501	32.70	1016860

### 3.3 Pharmacodynamic analysis of CM-VO in a mice CHD model

#### 3.3.1 Evaluation in the CHD model

**FIGURE 3:**
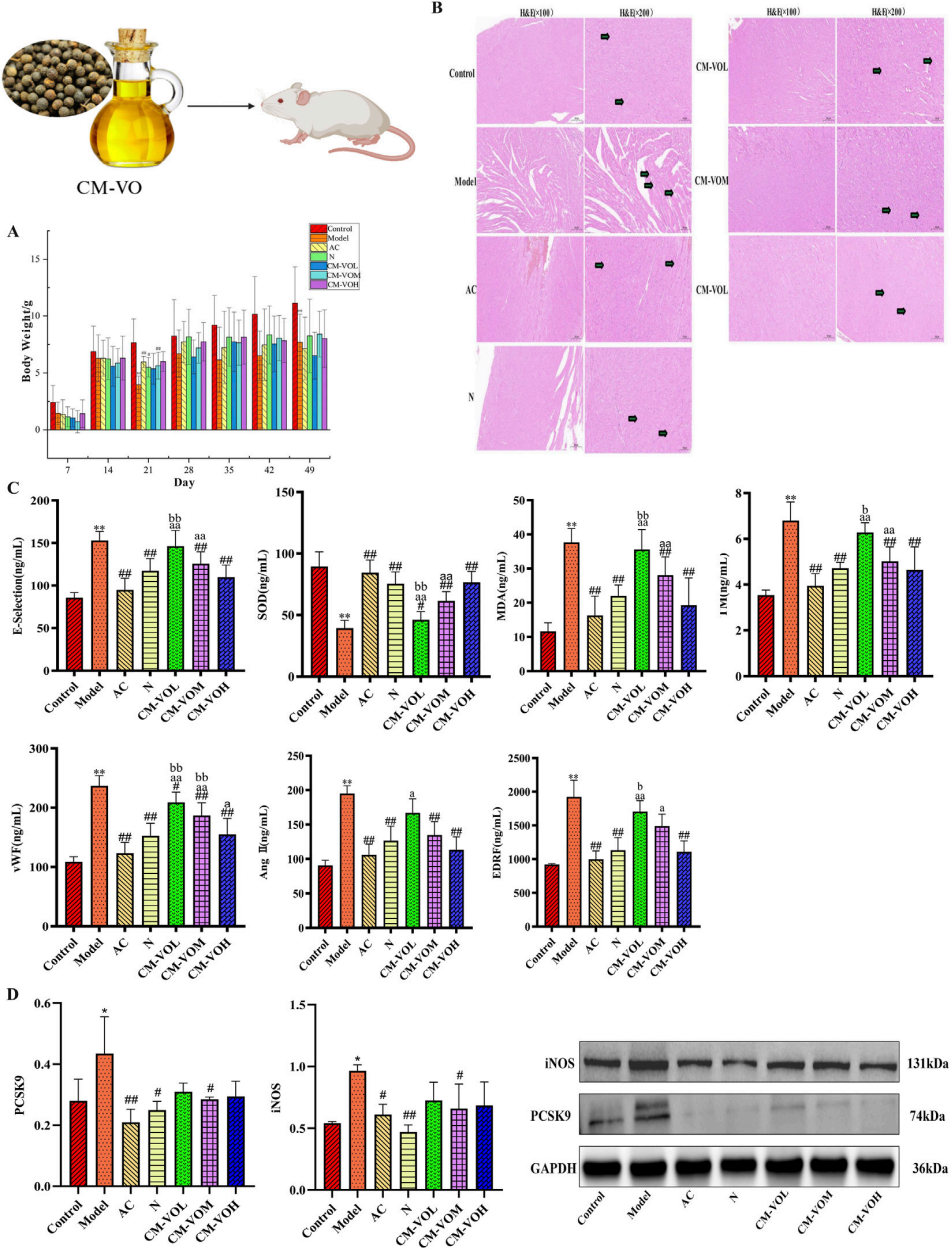
Pharmacodynamic study results chart. **(A)** The influence of CM-VO on the body weight of CHD mice (compared with the control group^*^ *P* < 0.05, ^*^ *P* < 0.01; compared with the model group ^#^ *P* < 0.05, ^#^ *P* < 0.01). **(B)** The staining images of myocardial tissues from CHD mice (left × 100, right × 200), the black arrow indicates lipids. **(C)** The expression level of E-selectin in mice serum (n = 6), compared with the control group, *P* < 0.01; compared with the model group, ^##^ *P* < 0.01; compared with the AC group, ^aa^ *P* < 0.01; compared with the N group, ^bb^ *P* < 0.01. The expression levels of MDA and SOD in mice serum (n = 6), compared with the control group, *P* < 0.01; compared with the model group, ^##^ *P* < 0.01; compared with the AC group, ^aa^ *P* < 0.01; compared with the N group, ^b^ *P* < 0.05, ^bb^ *P* < 0.01. The expression levels of TM and vWF in mice serum (n = 6), compared with the control group, ^**^ *P* < 0.01; compared with the model group, ^#^ *P* < 0.05, ^##^ *P* < 0.01; compared with the AC group, ^a^ *P* < 0.01, ^aa^ *P* < 0.01; compared with the N group, ^b^ *P* < 0.05, ^bb^ *P* < 0.01. The expression levels of Ang II and EDRF in mice serum (n = 6), compared with the control group, ^**^ *P* < 0.01; compared with the model group, ^#^ *P* < 0.05, ^##^ *P* < 0.01; compared with the AC group, ^a^ *P* < 0.05, ^aa^ *P* < 0.01; compared with the N group, ^b^ *P* < 0.05. **(D)** The expression levels of PCSK9 and iNOS in the cardiac tissues of CHD mice, A. control; B. model; C. AC; D. N; E. CM-VOL; F. CM-VOM; E. CM-VOH, compared with the control group, *P < 0.05; compared with the model group, ^#^ *P* < 0.05, ^##^ *P* < 0.01.

**FIGURE 4:**
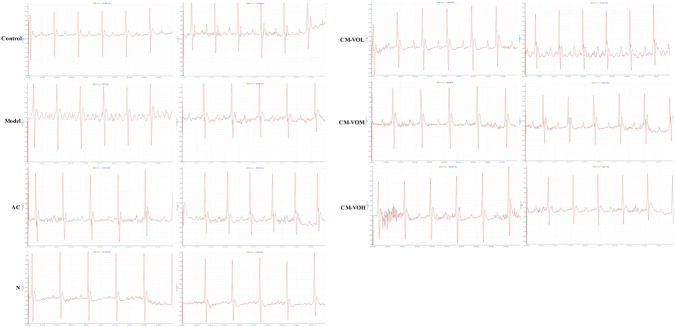
Effects of CM-VO on the electrocardiogram of CHD mice. Left: Before modeling; Right: After treatment.

This study systematically evaluated the multidimensional intervention effects of CM-VO on CHD mice. AC, N, and CM-VO had no significant impact on the body weight of CHD mice (Figure 3A). Research indicates that a contradiction exists between coronary blood supply and myocardial demand for blood, leading to increased myocardial tension and contractility, accelerated heart rate (Yuedai, 2013). The QTc interval, a heart rate-corrected QT interval, reflects the electrophysiological processes of ventricular depolarisation and repolarisation. When myocardial ischaemia, injury, or necrosis delays ventricular electrical activity, electrocardiograms exhibit QT interval prolongation. This prolonged state promotes atherosclerotic plaque deposition through mechanisms involving electromechanical coupling disruption and enhanced oxidative stress (Li et al., 2002). Electrocardiographic findings demonstrated that the AC, N, and CM-VOM groups significantly reduced heart rate while improving QTC interval and QRS duration (Figure 4).

#### 3.3.2 Histopathology

Myocardial histopathological examination revealed that CM-VO treatment mitigated myocardial fibre vacuolation and inflammatory cell infiltration, with the CM-VOH group exhibiting the most pronounced anti-inflammatory effect (Figure 3B).

#### 3.3.3 Serum biomarkers

Serological analysis confirmed that CM-VO dose-dependently reduced E-selectin expression to inhibit leukocyte-endothelial cell adhesion. It mitigated oxidative stress damage by enhancing the activity of SOD, and reducing MDA content. CM-VO significantly downregulated prothrombotic markers TM and vWF, as well as the vasoconstrictor Ang II, while improving vascular endothelial function (EDRF) levels (Figure 3C).

#### 3.3.4 Protein expression in myocardium

Western blot analysis revealed that, compared with the control group, the protein expression levels of both PCSK9 and iNOS were significantly upregulated in the cardiac tissue of the model group (P < 0.05). However, following intervention with CM-VO, the expression levels of PCSK9 and iNOS were markedly reduced (P < 0.05) (Figure 3D). These findings suggest that CM-VO may exert its anti-atherosclerotic effects, at least in part, through inhibition of the PCSK9/iNOS signaling pathway.

### 3.4 Network analysis-based mechanism prediction and transcriptomic target identification

#### 3.4.1 Active components and targets

**FIGURE 5:**
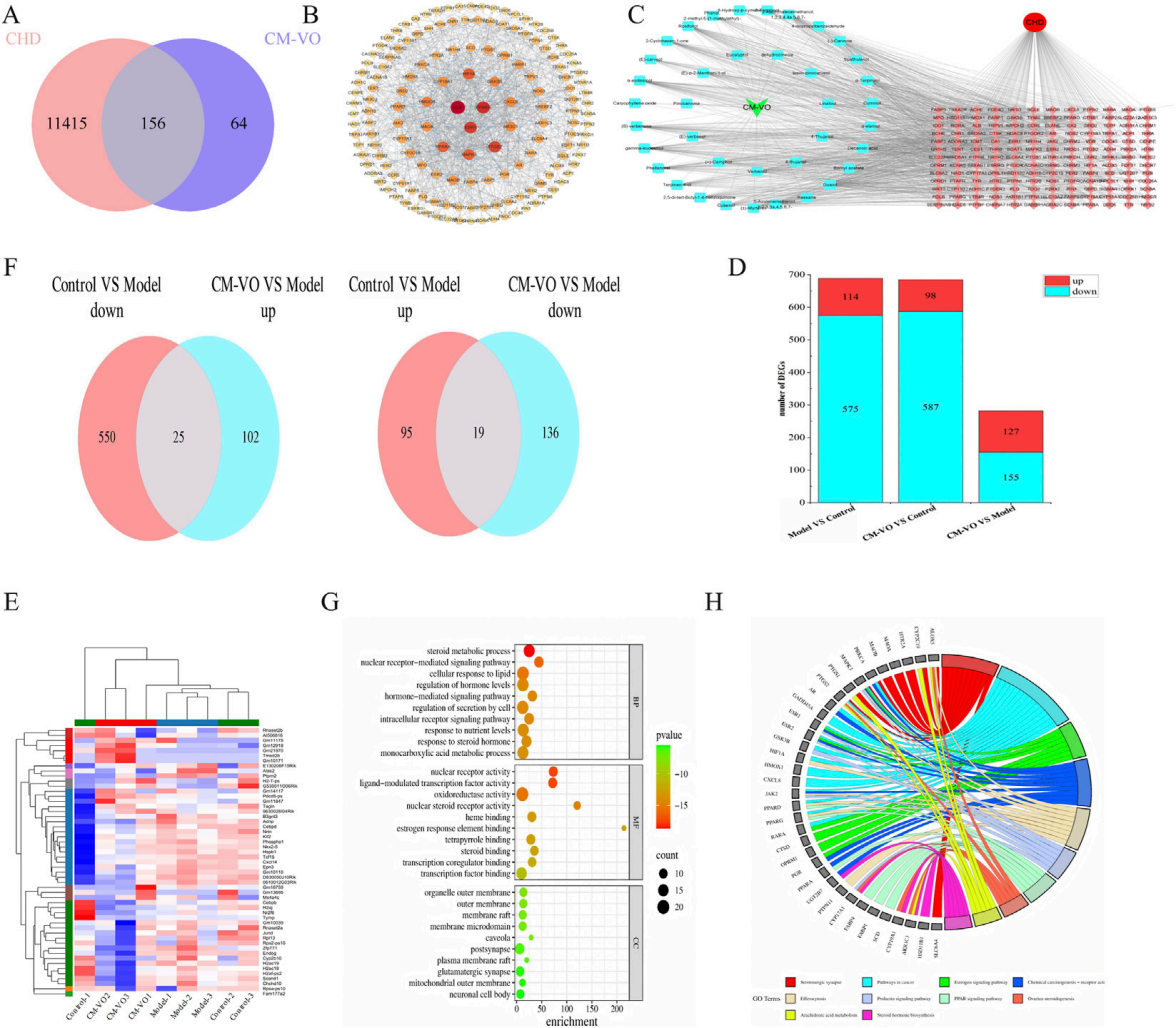
Network analysis and Transcriptomics result graph. **(A)** Venn diagram of CHD genes and CM-VO targets. **(B)** PPI protein interaction network diagram. **(C)** Component-Disease-Target network diagram. **(D)** Differentially expressed genes bar chart. (The horizontal axis represents the three comparison groups, the vertical axis denotes the number of differentially expressed genes, with red indicating upregulation and blue indicating downregulation). **(E)** Comparative clustering analysis of differentially expressed genes. **(F)** Gene-Wain Cross-Chart before and after CM-VO treatment. **(G)** GO enrichment analysis diagram for CM-VO treatment of CHD. **(H)** KEGG enrichment analysis diagram for CM-VO treatment of CHD.

A total of 39 active compounds were identified in CM-VO, including Eucalyptol, Linalool, α-Terpineol, and Carvacrol. Among these, Eucalyptol has been reported to promote cholesterol reverse transport, inhibit inflammatory cytokine release, and alleviate oxidative stress, thereby exerting anti-atherosclerotic effects (Savla and Bhatt, 2025). Linalool has demonstrated cardioprotective activity in myocardial infarction models, potentially through amelioration of oxidative stress via the Nrf2/HO-1 pathway, as well as suppression of apoptosis and inflammation (Mohamed et al., 2021). α-Pinene exhibits anti-inflammatory effects by reducing nitric oxide levels and myeloperoxidase activity (Khan et al., 2023). While Carvacrol has been shown to attenuate oxidative stress and reduce apoptotic cell death, suggesting therapeutic potential in cardiovascular diseases (Khazdair et al., 2024). Subsequent target prediction using the TCMSP and SwissTargetPrediction databases, followed by validation with UniProt, yielded 220 potential targets corresponding to these active compounds. Meanwhile, an integrated collection of 11,571 CHD-associated genes was compiled from multiple disease databases. The intersection of the compound-related targets and CHD-associated genes revealed 156 overlapping targets (Figure 5A), which represent the potential therapeutic targets through which the active components of CM-VO may exert protective effects against CHD.

#### 3.4.2 PPI Network

The 156 common targets were input into the String database to obtain target protein interaction network data. Visualisation was performed using Cytoscape 3.9.1 (Figure 5B). Based on the median values of degree, BC and CC as nodes, 58 key targets were identified, providing a preliminary reference for future researchers. Concurrently, a component-disease-target visualisation network diagram was created using component, disease, and target data (Figure 5C).

#### 3.4.3 Transcriptomic identification of DEGs

Transcriptome sequencing of nine samples yielded 59.19 Gb of clean data with high quality. Differentially expressed genes (DEGs) were filtered using FDR < 0.05 and |log_2_FC| ≥ 1: 689 DEGs were identified in the model vs. control group, and 685 in the CM-VO vs. control group. Among these, 98 genes were upregulated and 587 were downregulated. CM-VO vs. model group yielded 282 DEGs, comprising 127 upregulated and 155 downregulated genes (Figure 5D). Cluster analysis revealed distinct expression patterns within each group (Figure 5E). A total of 44 genes exhibited opposite expression trends following modelling and treatment: 25 genes were downregulated after modelling but upregulated after treatment, while 19 genes were upregulated after modelling but downregulated after treatment (Figure 5F). By integrating transcriptomic profiles with the core targets derived from network pharmacology, we identified 101 promising genes, which were further subjected to functional enrichment analysis to assess their biological relevance. This joint enrichment approach was designed to determine whether the computationally predicted targets and experimentally responsive genes converged on biological processes associated with coronary heart disease (CHD), rather than to serve as the basis for selecting the final validation targets.

#### 3.4.4 GO enrichment

Notably, these genes were significantly enriched in key biological processes, including the regulation of hormone levels and cellular secretion. In addition, they were associated with critical molecular functions such as oxidoreductase and nuclear receptor activity, and were localized to cellular components like receptor complexes and the organelle outer membrane (Figure 5G).

#### 3.4.5 KEGG enrichment

KEGG pathway analysis indicated that the forward genes were primarily involved in pathways including chemical carcinogen receptor activation, serotonergic synapse, arachidonic acid metabolism, lipids and atherosclerosis, and hepatitis B (Figure 5H).

### 3.5 Deep learning model performance and molecular docking

#### 3.5.1 Data preparation

Based on the 44 differentially expressed genes (DEGs) identified in a previous transcriptomic study on the treatment of coronary heart disease (CM-VO), the official gene symbols for these genes were obtained by setting the species filter in the UniProt database to ‘human’ and performing a cross-reference. This process resulted in the identification of 19 human genes, which were ultimately selected as inputs for the encoder (Table 3).

**TABLE 3:** Transcriptomic differential genes of CM-VO in the treatment of CHD.

Sequence number	Full name	Gene name	Uniprot
1	Growth arrest and DNA damage-inducible protein alpha	GADD45A	P24522
2	C-C chemokine receptor type 2	CCR2	P41597
3	Tripartite motif containing 7	TRIM7	Q9C029
4	Helt bHLH transcription factor	HELT	A6NFD8
5	Glucocorticoid induced 1	GLCCI1	Q86VQ1
6	5‘-aminolevulinate synthase 2	ALAS2	P22557
7	ChaC glutathione specific gamma-glutamylcyclotransferase 1	CHAC1	Q9BUX1
8	Synuclein alpha	SNCA	P37840
9	Alpha kinase 1	ALPK1	Q96QP1
10	Ribosomal protein S20	RPS20	P60866
11	Myelin protein zero like 2	MPZL2	O60487
12	Butyrophilin like 2	BTNL2	Q9UIR0
13	RasGEF domain family member 1B	RASGEF1B	Q0VAM2
14	Tudor and KH domain containing	TDRKH	Q9Y2W6
15	Methenyltetrahydrofolate synthetase	MTHFS	P49914
16	Ribosomal protein S27	RPS27	P42677
17	Plasmalemma vesicle associated protein	PLVAP	Q9BX97
18	Ribosomal protein L14	RPL14	P50914
19	Ribosomal protein L22 like 1	RPL22L1	Q6P5R6

#### 3.5.2 CM-VO blood component information

The CM-VO components entering the bloodstream, identified through serum pharmacokinetic studies, were mapped to their corresponding candidate parent compounds based on serum metabolite source inference, pathway enrichment (arachidonic acid metabolism), and structural similarity analysis. The chemical structures of these candidate compounds were then downloaded from the PubChem database. Their PubChem-ID and SMILES information were input into the decoder (Table 4).

**TABLE 4:** CM-VO blood component information.

Sequence number	Chemical name	Pubchem-ID	Smiles
1	*α*-pinene	6654	CC1=CCC2CC1C2(C)C
2	*β*-pinene	440967	CC1(C2CCC(=C)C1C2)C
3	Decanoic acid	2969	CCCCCCCCCC(=O)O
4	*β*-myrcene	31253	CC(=CCCC(=C)C=C)C
5	Linalool	6549	CC(=CCCC(C)(C=C)O)C

#### 3.5.3 Affinity prediction

**FIGURE 6:**
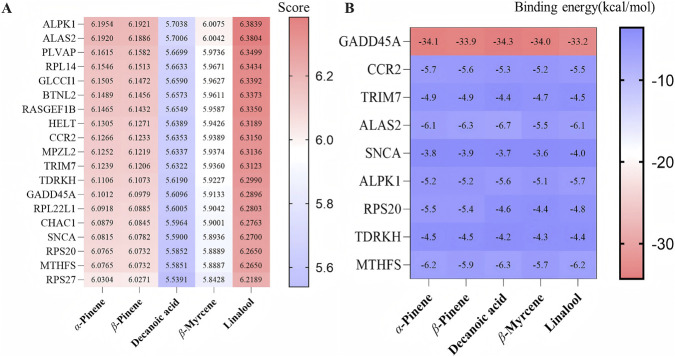
Deep Learning scoring graph. **(A)** Blood-binding component - Transcriptional target affinity score. **(B)** Blood-binding components - Transcriptional target molecule docking results.

Following evaluation of the four regression models, the gradient-boosted regression model demonstrated optimal performance with the lowest mean squared error and a coefficient of determination closest to 1, and was therefore selected for subsequent predictions. The model scores the affinity between CM-VO blood components and transcriptional targets, ultimately producing a visualisation (Figure 6A).

#### 3.5.4 Molecular docking

Binding energies below 0 kcal/mol indicate spontaneous interactions, while values below −5.0 kcal/mol suggest strong binding affinity (Gao et al., 2025). All compound–protein pairs showed binding energies below −5 kcal/mol (Table 5), indicating stable interactions, with GADD45A, MTHFS, and ALAS2 ranking highest. Docking conformations revealed that binding stability was mainly driven by hydrogen bonding and hydrophobic interactions. Hydrogen bonds stabilized ligand orientation, while hydrophobic contacts enhanced binding of non-polar regions. Key residues in GADD45A, MTHFS, and ALAS2 participated in ligand binding through multiple non-covalent interactions, including hydrogen bonds and hydrophobic contacts (Figure 6B). GADD45A showed a more compact binding pocket and higher interaction density, possibly explaining its stronger affinity. Overall, ligand–protein complexes were stabilized by hydrogen bonding, hydrophobic interactions, and key residue contacts.

**TABLE 5:** Score results and visualization analysis.

Gene name	Structure	Protein	Molecular docking	Docking score (kcal/mol)	Affinity score
*α*-pinene	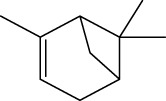	GADD45A	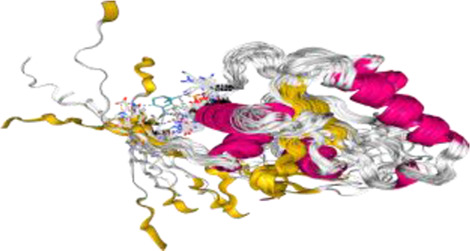	−34.1	6.1012
		MTHFS	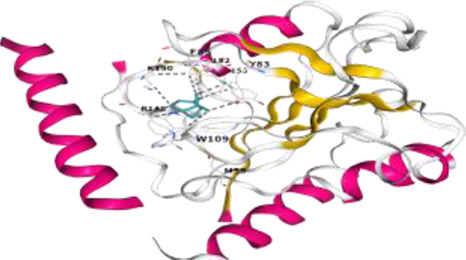	−6.2	6.0765
		ALAS2	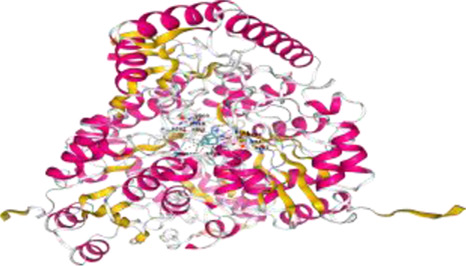	−6.1	6.1920
		GADD45A	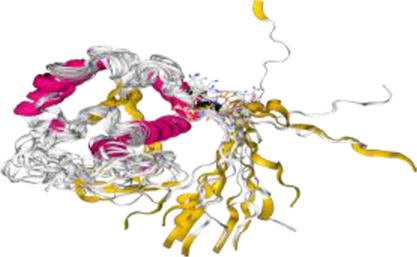	−33.9	6.0979
*β*-pinene	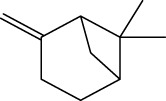	MTHFS	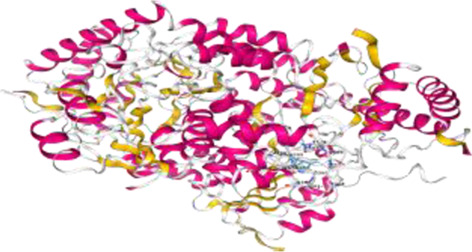	−6.3	6.0732
		ALAS2	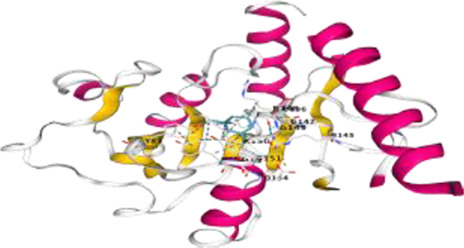	−5.9	6.1886
		GADD45A	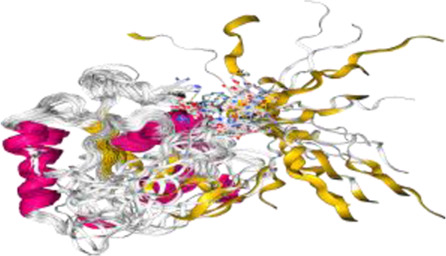	−34.3	5.6096
Decanoic acid	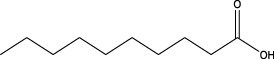	MTHFS	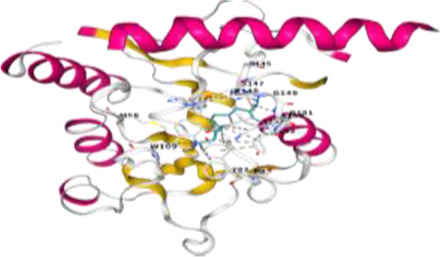	−6.7	5.5851
		ALAS2	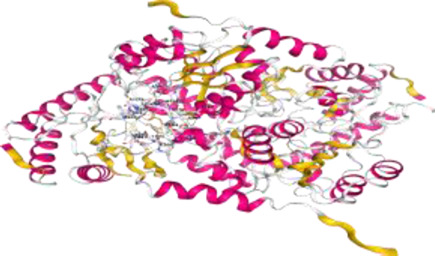	−6.3	5.7006
		GADD45A	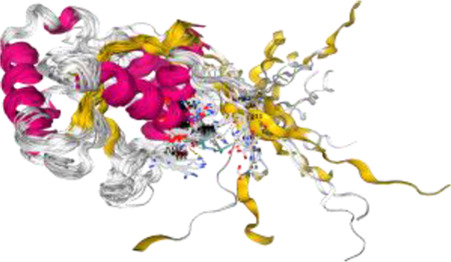	−34	5.9133
*β*-myrcene	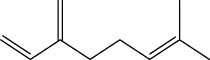	MTHFS	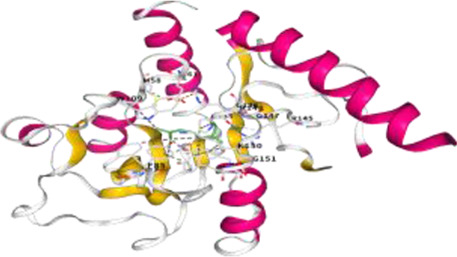	−5.7	5.8887
		ALAS2	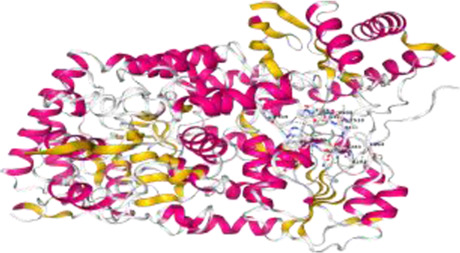	−5.5	6.0042
		GADD45A	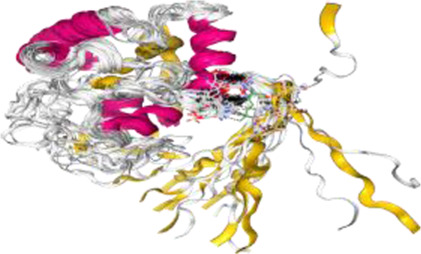	−33.2	6.2896
Linalool	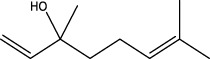	MTHFS	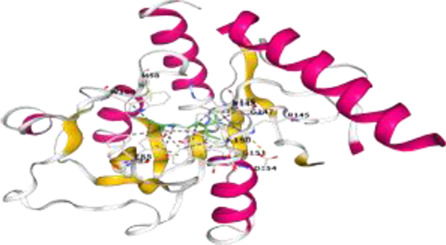	−6.2	6.265
		ALAS2	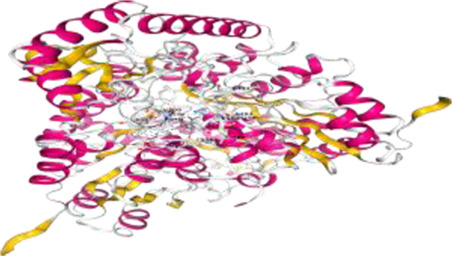	−6.1	6.3804

#### 3.5.5 Comprehensive analysis

Integrating machine learning affinity scores with molecular docking binding energy results, GADD45A, MTHFS, and ALAS2 were identified as the top three key target proteins (Table 5). Literature review confirmed their association with cardiomyopathies, providing theoretical basis and support for subsequent experiments.

### 3.6 Protective effects of CM-VO-containing serum on H9c2 cells

#### 3.6.1 Serum concentration effect on viability

**FIGURE 7:**
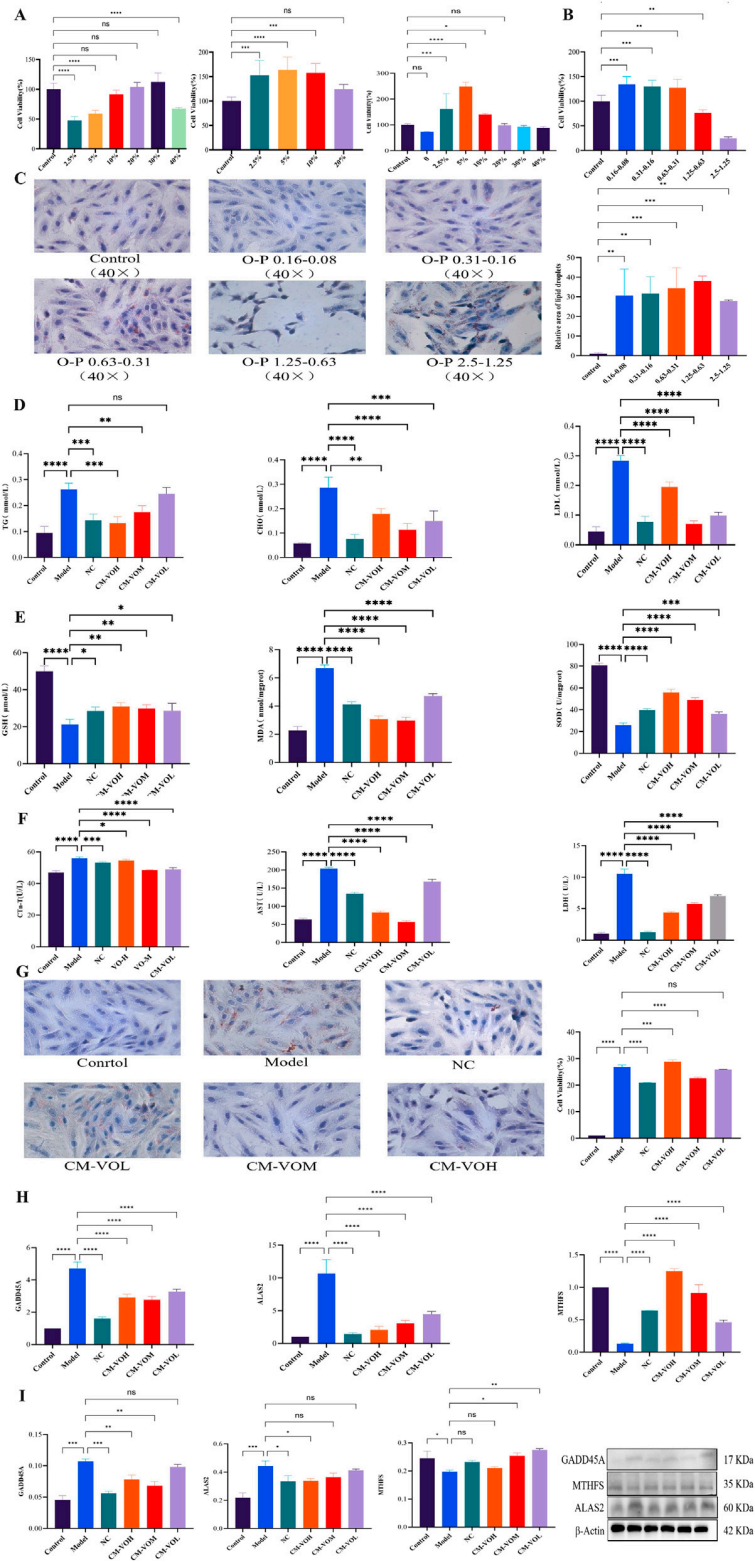
Cell Experiment verification diagram. **(A)** The results of the screening for the optimal serum addition amount. **(B)** The results of the effects of different concentrations of O-P on the viability of H9c2 cardiac muscle cells. **(C)** The results of the effects of different concentrations of O-P on lipid accumulation in H9c2 cardiomyocytes (Left: Oil Red O staining image; Right: Quantitative analysis of lipid accumulation using Image J). **(D)** The effects of different volumes of CM-VO drug-containing serum on lipid damage in H9c2 cardiac muscle cells stimulated by O-P (TG; CHO; LDL). **(E)** The effects of different volumes of CM-VO drug-containing serum on oxidative stress injury in O-P stimulated H9c2 cardiomyocytes (GSH; SOD; MDA). **(F)** The effects of different volumes of CM-VO drug-containing serum on myocardial injury caused by O-P stimulation in H9c2 cardiomyocytes (CTn-T; AST; LDH). **(G)** The effects of different volumes of CM-VO drug-containing serum on the viability and lipid accumulation of H9c2 cardiomyocytes stimulated by O-P (Left: Oil Red O staining diagram; Right: Quantitative analysis of lipid accumulation using Image J). **(H)** The relative expression levels of GADD45A, MTHFS, and ALAS2 mRNA in H9c2 cells (n = 9). **(I)** Western blot was used to detect the expression levels of GADD45A, MTHFS and ALAS2 proteins in cardiac muscle cells.

Experimental results indicate that the optimal addition rate for blank serum is 20%, while drug-containing serum for positive controls is 5%. Drug-containing CM-VO serum significantly enhances H9c2 cell viability within the range of 2.5%–10% (Figure 7A).

#### 3.6.2 O-P induced lipid toxicity model

Using O-P 1.25–0.63 mmol·L^−1^ for 12 h successfully established a cellular lipotoxicity damage model (Figure 7B), characterised by decreased cell viability and increased lipid accumulation (Figure 7C). Concurrently, model group cells exhibited elevated intracellular TG, CHO, and LDL levels (Figure 7D), reduced oxidative stress markers GSH and SOD, increased MDA (Figure 7E), and elevated myocardial injury markers CTn-T, AST, and LDH (Figure 7F).

#### 3.6.3 Effects of CM-VO serum on key targets

Following intervention with CM-VO drug-containing serum, these pathological alterations were markedly ameliorated, with reduced lipid accumulation and restoration of relevant indicators (Figure 7G). At the molecular level, O-P stimulation upregulated GADD45A and ALAS2 mRNA and protein expression while downregulating MTHFS expression (Figure 7H). Following CM-VO intervention, GADD45A and ALAS2 protein expression decreased, while MTHFS expression increased in the group (Figure 7I).

## 4 Discussion

This study identified 68 constituents in CM-VO via GC-MS, primarily monoterpenes and sesquiterpenes. Serum pharmacochemistry identified eight bioavailable metabolites. Components like α-pinene and linalool, also found in CM-VO, are known for cardiovascular protective effects, such as inhibiting NF-κB-mediated inflammation (Zhang et al., 2020) and enhancing antioxidant capacity (Oner et al., 2019).

*In vivo*, CM-VO improved ECG parameters (QTc, QRS) and reduced myocardial inflammation, showing efficacy comparable to atorvastatin (lipid-lowering) (Kang et al., 2013) and nicorandil (vasodilation) (Tarkin and Kaski, 2016). Notably, CM-VO uniquely regulated vascular endothelial function by downregulating prothrombotic factors (TM, vWF) and Ang II while upregulating EDRF, suggesting a combined “lipid-lowering, anti-inflammatory, and endothelial-protective” mechanism.

Oxidative stress and inflammation are key drivers of atherosclerosis (Ajoolabady et al., 2024). CM-VO dose-dependently enhanced SOD, reduced MDA, and suppressed E-selectin and iNOS. Significantly, it downregulated PCSK9, a core statin target involved in LDL receptor degradation (Ferrari et al., 2019). This indicates CM-VO can engage key modern drug pathways while leveraging TCM’s multi-target advantage, offering a potential integrative treatment approach.

Based on the 68 chemical constituents of CM-VO identified by GC–MS, network analysis was performed to predict the potential compound–target–disease interaction network associated with coronary heart disease, resulting in the identification of 39 bioactive compounds and 58 key targets. This analysis provided a global view of the multi-component and multi-target characteristics of CM-VO. In parallel, transcriptomic analysis identified 44 treatment-responsive DEGs in the CHD model. The transcriptomic DEGs and network-derived core targets were further jointly subjected to enrichment analysis, yielding 101 disease-associated genes to evaluate whether the computationally predicted targets and experimentally responsive genes converged on CHD-related biological processes, rather than to define the final validation targets. Subsequently, the 44 DEGs were standardized to 19 human genes using UniProt and evaluated in relation to serum-related candidate bioavailable components. Deep learning and molecular docking analyses ultimately prioritized GADD45A, MTHFS, and ALAS2 as high-affinity protein targets. Overall, this integrated strategy supports the pharmacological basis and potential anti-CHD mechanisms of CM-VO from complementary perspectives, including full-component network prediction, serum pharmacochemistry, transcriptomic response, and computational target validation.

The three core targets identified in this study—GADD45A, MTHFS and ALAS2—correspond to the three major pathological modules of inflammation and apoptosis, folate metabolism, and iron homeostasis, respectively. Together, they constitute the key molecular network underlying the CM-VO intervention for coronary heart disease, demonstrating clear clinical relevance and translational potential. GADD45A (growth arrest and DNA damage-inducible alpha) is significantly overexpressed during myocardial ischaemia–hypoxia. By activating the p38 MAPK pathway, it promotes cardiomyocyte apoptosis and the release of inflammatory factors, thereby exacerbating myocardial injury (Liu et al., 2021). Clinical studies have confirmed that GADD45A mRNA levels are significantly elevated in the aortic adventitia of patients with coronary heart disease (Oma et al., 2018); moreover, its expression is positively correlated with markers of myocardial damage, inflammation and disease severity, and may serve as a potential biomarker for myocardial ischaemic injury (Wang et al., 2022). In the present study, CM-VO-containing serum significantly downregulated GADD45A expression, suggesting that it may block ischaemia-induced myocardial apoptosis and inflammatory responses by inhibiting the GADD45A/p38 MAPK pathway, thereby offering a novel target for protecting the myocardium against ischaemic damage. MTHFS (5,10-methenyltetrahydrofolate synthetase) is involved in folate metabolism; folate deficiency leads to elevated homocysteine (Hcy) levels, which exacerbate vascular endothelial injury and atherosclerosis (Jolivet et al., 1996). Clinical studies have demonstrated that plasma homocysteine is indeed significantly higher in patients with coronary heart disease than in healthy individuals, and that higher levels are associated with more severe vascular lesions (Bosevski et al., 2020). Our results show that CM-VO significantly upregulates MTHFS expression, suggesting that it may provide a new intervention strategy for hyperhomocysteinaemia-associated CHD by improving folate metabolism, lowering Hcy levels, correcting metabolic disturbances and protecting the vascular endothelium. ALAS2 (5-aminolevulinate synthase 2) is a key enzyme in haem synthesis; its aberrant activation causes iron overload, which in turn generates large amounts of reactive oxygen species (ROS) via the Fenton reaction, thereby exacerbating oxidative stress-induced myocardial injury (He et al., 2024). Clinical evidence indicates that oxidative stress elevates ROS levels and impairs antioxidant defence mechanisms, a phenomenon that is particularly pronounced in patients with coronary artery disease (Lubrano and Balzan, 2015). In this study, CM-VO significantly downregulated ALAS2 expression, suggesting that it may alleviate myocardial lipid peroxidation and cellular damage by inhibiting ALAS2-mediated iron overload and oxidative stress, thus providing a new therapeutic target for oxidative stress-related injury in coronary heart disease.

This study innovatively applied deep learning (RDKit, ProtBERT) to improve affinity prediction accuracy. Validation used a comprehensive “in vivo-in vitro” strategy. The *in vitro* model employed O-P-induced lipotoxicity in H9c2 cells, simulating cardiomyocyte damage in CHD. Using drug-containing serum for intervention better approximates the *in vivo* pharmacokinetic environment, enhancing translational relevance and conclusion reliability.

## 5 Conclusion

Employing a multidisciplinary strategy integrating multi-omics and deep learning, this study systematically reveals that CM-VO exerts comprehensive anti-CHD effects—including anti-inflammatory, antioxidant, vascular endothelial regulation, and lipid metabolism modulation—through multi-component, multi-target mechanisms involving core protein targets like GADD45A, MTHFS, and ALAS2. These findings provide a scientific rationale for the clinical application and secondary development of CM-VO and demonstrate the utility of integrated omics approaches in elucidating TCM mechanisms.

## Data Availability

The datasets presented in this study can be found in online repositories. The names of the repository/repositories and accession number(s) can be found in the article/Supplementary Material.
