# Supplementary material for: Integrating multi-omics and deep learning to explore the active ingredients and molecular mechanisms of Cinnamomum migao volatile oil in the treatment of coronary heart disease
Source: Front Pharmacol. 2026 Aug 5;17:1823482. doi: 10.3389/fphar.2026.1823482 (PMC13486190; doi:10.3389/fphar.2026.1823482)
Supplement: Supplementary file 3 [file Supplementaryfile1.docx]

Appendix

Table A. Materials and Reagents Information Table

| Reagent Name | Manufacturer | Batch Number or Item Number |
| --- | --- | --- |
| Large-fruited Galangal | Luodian Town, Luodian County, Guizhou Province |  |
| Atorvastatin Calcium Tablets | Lepu Pharmaceutical Technology Co., Ltd. | 202211120B |
| Nicardipine Tablets | Xi'an Hanfeng Pharmaceutical Co., Ltd. | 2302051 |
| E-Selective Reagent Kit | Jiangsu Jingmei Biotechnology Co., Ltd. | 20231011009 |
| Glutaraldehyde Test Kit | Jiangsu Jingmei Biotechnology Co., Ltd. | 20231018003 |
| Superoxide Dismutase Assay Kit | Jiangsu Jingmei Biotechnology Co., Ltd. | 20231016031 |
| Thrombomodulin Assay Kit | Jiangsu Jingmei Biotechnology Co., Ltd. | 20230913028 |
| Vascular Haemophilia Factor Test Kit | Jiangsu Jingmei Biotechnology Co., Ltd. | 20230908016 |
| Endothelium-Dependent Relaxin Factor Assay Kit | Jiangsu Jingmei Biotechnology Co., Ltd. | 20230830062 |
| Angiotensin II Assay Kit | Jiangsu Jingmei Biotechnology Co., Ltd. | 20231010022 |
| GAPDH | Wuhan Sanying Biotechnology Co., Ltd. | 10028230 |
| iNOS | Wuhan Sanying Biotechnology Co., Ltd. | 00121680 |
| PCSK9 | Wuhan Sanying Biotechnology Co., Ltd. | 00105615 |
| HPR-conjugated Affinipure Goat Anti-Mouse IgG (High Avidity) | Wuhan Sanying Biotechnology Co., Ltd. | 20000838 |
| HPR-labelled Goat Anti-Mouse/Rabbit IgG | Shanghai Yamei Biopharmaceutical Technology Co., Ltd. | 026A1115 |
| PMSF | Aladdin Reagent Co., Ltd. | P105539 |
| RIPA Lysis Buffer | Biyuntian Biological Reagent Co. | P0013B |
| BCA Protein Concentration Assay Kit | Biyuntian Biological Reagent Company | P0010 |
| 10% PAGE Coloured (Red) Gel Super-Fast Preparation Kit | Sinopharm Chemical Reagents Co., Ltd. | 80125336 |
| Prestained Protein Marker VII  (8-195 kDa) | Wuhan Savier Biotechnology Co., Ltd. | G2087-250μL |
| Ultra-Sensitive ECL Chemiluminescence Assay Kit | Dalian Meilun Biotechnology Co., Ltd. | MA0186-1 |
| SWE Rapid High-Resolution Electrophoresis Buffer | Wuhan Savier Biotechnology Co., Ltd. | G2149-1L |
| Ice-Free Rapid Transfer Buffer | Wuhan Savier Biotechnology Co., Ltd. | G2148-1L |
| TBST Buffer | Wuhan Savier Biotechnology Co., Ltd. | G2150-1L |
| Primary Antibody Dilution Buffer | Wuhan Savier Biotechnology Co., Ltd. | G2025-100ML |
| Western Blot Secondary Antibody Dilution Buffer | Wuhan Savier Biotechnology Co., Ltd. | G2009-100ML |
| Anhydrous Ethanol | Sinopharm Chemical Reagent Co., Ltd. | 100092683 |
| Xylene | Sinopharm Chemical Reagent Co., Ltd. | 10023418 |
| n-Butanol | Sinopharm Chemical Reagents Co., Ltd. | 100052190 |
| Environmentally Friendly Dewaxing Solution | Wuhan Saville Biotechnology Co., Ltd. | G1128 |
| Universal Tissue Fixative | Wuhan Saviell Biotechnology Co., Ltd. | G1101 |
| Hematoxylin and Eosin (H&E) High-Definition Permanent Staining Kit | Wuhan Savier Biotechnology Co., Ltd. | G1076 |
| Neutral Gum | Sinopharm Chemical Reagent Co., Ltd. | 10004160 |
| NC Membrane 0.45 μm | BioTrace | 71151558 |
| Sodium Carboxymethyl Cellulose, Standard Grade | Shanghai Changguang Enterprise Development Co., Ltd. |  |
| MJZol Total RNA Extraction Kit | Shanghai MJ Bio-Pharmaceutical Technology Co., Ltd. (China) |  |
| RNA Purification Kit | Shanghai Meiji Biopharmaceutical Technology Co., Ltd. (China) |  |
| Biowest Agarose | Biowest (Spain) |  |
| Illumina® Stranded mRNA Prep, Ligation (High Throughput) | Illumina (USA) |  |
| NovaSeq Reagent Kit | Illumina (USA) |  |
| Saturating Oil Red O Stain | Servicebio | CR2305087/G1015 |
| Nicorandil | Aladdin | N129566 |
| Bovine Serum Albumin BSA Domestic | Servicebio | GC2405110/GC305010-50g |
| Palmitic Acid | Solarbio | 24231231004/H8780 |
| Oleic acid | Solarbio | 142405170003/O8291 |
| CCK-8 assay kit | TargetMol | P24072600911/C0005 |
| BCA Protein Quantification Kit | Servicebio | CR2403100-1/G2026 |
| Total Superoxide Dismutase (T-SOD) Detection Kit | Servicebio | MPC2411029/G4306 |
| Malondialdehyde (MDA) Test Kit | Servicebio | MPC2406080/G4300 |
| Reduced Glutathione (GSH) Detection Kit | Servicebio | MPC2405067/G4305 |
| Aspartate Aminotransferase (AST) Assay Kit | Rayto | 20240715/S03040 |
| Triglyceride (TG) Assay Kit | Rayto | 20240822/S03027 |
| Total Cholesterol (CHO) Assay Kit | Rayto | 20240912/S03042 |
| Low-Density Lipoprotein (LDL) Assay Kit | Rayto | 20240626/S03029 |
| Lactate Dehydrogenase (LDH) Assay Kit | Rayto | 20240827/S03034 |
| Rat Cardiac Troponin T (CTn-T) ELISA Research Kit | Servicebio | 20241216/J40097-A |
| GADD45A | ABclonal | 5500029744/A1797 |
| MTHFS | ABclonal | 0109260201/A15504 |
| ALAS2 | ABclonal | 0020740201/A6522 |
| FCER1G | ABclonal | 0060020101/A12889 |
| RPLP1 | ABclonal | 0026140201/A6725 |
| LIMD2 | Proteintech | 00082585/15471-1-AP |
| HPR-labelled goat anti-rabbit | Servicebio | AC2406022825/GB23303 |
| RIPA Lysis Buffer | Servicebio | CR2409059/G2002 |
| 10% PAGE Coloured (Red) Gel Super-Fast Preparation Kit | Vazyme | 7E2380K4/E303-01 |
| Prestained Protein Marker VIII  (8-270 kDa) | Servicebio | MPC2410088/G2089 |
| Ultra-Sensitive ECL Chemiluminescence Assay Kit | Meilunbio | MA0186-Oct-14J/MA0186-1 |
| SWE Rapid High-Resolution Electrophoresis Buffer | Servicebio | GA2405102/G2081-1L |
| Ice-Free Rapid Transfer Buffer | Servicebio | GA2409101/G2081-1L |
| 10× TBST Buffer | Servicebio | GC2405001/G0004-1L |
| β-Actin Mouse Monoclonal Antibody | Epizyme | 028B1012/LF201S |

Table B. Experimental Instruments and Equipment Information

| Name | Manufacturer | Model |
| --- | --- | --- |
| Biological Signal Acquisition and Analysis System | Chengdu Taimeng Software Co., Ltd. | BL-420 |
| Dehydrator | DIAPATH | Donatello |
| Embedding Machine | Wuhan Junjie Electronics Co., Ltd. | JB-P5 |
| Tissue Micro-sectioning Machine | Shanghai Leica Instruments Co., Ltd. | RM2016 |
| Freezing Stage | Wuhan Junjie Electronics Co., Ltd. | JB-L5 |
| Tissue Spreader | Zhejiang Jinhua Kedi Instrument Equipment Co., Ltd. | KD-P |
| Oven | Tianjin Laiborui Instrument Equipment Co., Ltd. | GFL-230 |
| Cryostat | Thermo Fisher Scientific (China) Co., Ltd. | CRYOSTAR NX50 |
| Adhesive Slides (Paraffin Sections) (White Lacquer Tape) | Wuhan Savier Biotechnology Co., Ltd. | G6012-1 |
| Adhesive Microscope Slides (Frozen Sections) (White Painted Ribbon) | Wuhan Savier Biotechnology Co., Ltd. | G6012-2 |
| Enzyme-Linked Immunosorbent Assay (ELISA) Reader | Shenzhen Leidu Life Sciences Co., Ltd. | Rayto RT-6100 |
| Chemiluminescence Analyzer | CLINX | 6100 |
| Handheld Homogeniser | Shanghai Lichenbangxi Instrument Technology Co., Ltd. | LC-SFJ-10 |
| Centrifuge | Yancheng Kate Laboratory Instrument Co., Ltd. | TG18G |
| Power Supply Instrument Power Supply | Shanghai Jiapeng Technology Co., Ltd. | Keebio-600N |
| Metal Bath | Qun'an Laboratory Instruments Co., Ltd. | MDB100 |
| Horizontal Rotary Shaker | Taizhou Nuomi Medical Technology Co., Ltd. | NMSP-600 |
| Vortex Mixer | Changzhou Yuexin Instrument Manufacturing Co., Ltd. | XH-C |
| Palm Centrifuge | Wuhan Savier Biotechnology Co., Ltd. | MS6000 |
| Agilent HP6890/5975C Gas Chromatograph | Agilent Technologies (USA) |  |
| Electric Heating Mantle | Tianjin Test Instrument Co., Ltd. | 98-1-B |
| Volatile Oil Detector | Anhui Meixing Laboratory Equipment Co., Ltd. | HAD-1785 |
| Grinding Mill | Shanghai Wanbai Biotechnology Co., Ltd. | TL-48R |
| Compact Centrifuge | [Hefei ABSON Scientific Instruments Co., Ltd.](http://www.baidu.com/link?url=HRD2f5dd4tugJJvg26_1VGDP8QV3k6XNHZt2uttlRr65XrOpWSVBRrFT-DPuJ6mY) | ABSON MiFly-6 |
| High-Speed Desktop Refrigerated Centrifuge | Eppendorf | Eppendorf 5424R |
| Electrophoresis Unit | Beijing Liuyi Instrument Factory | DYY-6C |
| Micro-volume Spectrophotometer | Thermo Fisher Scientific | NanoDrop2000 |
| Bioanalyser | Agilent | Agilent 5300 |
| Fluorescence Quantifier | thermofisher | Qubit 4.0 |
| PCR machine | BIO-RAD | T100 Thermal Cycler |
| Sequencer | Illumina | NovaSeq 6000 |
| Vertical Refrigerated Display Cabinet | Xingxing | LSC-316C |
| Horizontal Freezer | Meiling | BCD-318AT |
| Electronic Balance | Mettler Toledo Instruments (Shanghai) Co., Ltd. | ME203E/02 |
| High-Speed Tissue Grinder | Servicebio | KZ-III-F |
| High-Speed Low-Temperature Centrifuge | Servicebio | SLX-1024F |
| Electric Heating Constant Temperature Water Bath | Jinghong | DK-8B |
| Handheld Centrifuge | Servicebio | MC-700 |
| Enzyme-Linked Immunosorbent Assay Reader | BioTeK | Epoch |
| Fully Automated Biochemical Analyser | Rayto | Chemray 240 |
| Clean Bench | Suzhou Antai Air Co., Ltd. | SW-CJ-2F |
| Carbon Dioxide Incubator | Thermo | YZB/USA |
| Inverted Fluorescence Microscope | Shanghai Lai's Electronic Technology Co., Ltd. | CKX53 |
| Agilent Gas Chromatography-Single Quadrupole Mass Spectrometer | USA | 19091S-436 |
| Nitrogen Blower | Tianjin Autocell | MTN-2800D |
| Electric Heating Constant Temperature Industrial Water Bath | Tianjin Test Instrument Co., Ltd. | SHHW21.600AI |
| Vortex Mixer | Shanghai Yidian Scientific Instruments Co., Ltd. | HY-2 |

Table C. The composition and relative percentage content of the CM-VO

| Peak | Full Name | Chemical Name | Molecular Formula | Exact Mass | RT | Area | Classification | *MS/MS* | Reference |
| --- | --- | --- | --- | --- | --- | --- | --- | --- | --- |
| 1 | Bicyclo[3.1.0]hex-2-ene, 4-methyl-1-(1-methylethyl)- | β-Thujene | C_10_ H_16_ | 136.1252 | 11.82 | 75172635 | Monoterpenes | 136.1246,  121.1011,  93.0698,  92.0620,  77.0385,  65.0385,  53.0385,  41.0385 | [1] |
| 2 | α-Pinene | α-Pinene | C_10_H_16_ | 136.1252 | 12.09 | 597791369 | Monoterpenes | 136.1246,  121.1011,  105.0698,  93.0698,  92.0620,  79.0542,  94.0733,  41.0385,  43.0542 | [1] |
| 3 | Camphene | Camphene | C_10_ H_16_ | 136.1252 | 12.51 | 64495310 | Monoterpenes | 136.1246,  121.1011,  107.0855,  95.0855,  93.0698,  79.0542,  67.0542,  41.0385 | [1] |
| 4 | Bicyclo[3.1.0]hexane, 4-methylene-1-(1-methylethyl)- | Sabinene | C_10_ H_16_ | 136.1252 | 13.19 | 676816690 | Monoterpenes | 136.1246,  121.1011,  93.0698,  91.0542,  79.0542,  77.0385,  92.0620 | [1] |
| 5 | Bicyclo[3.1.1]heptane, 6,6-dimethyl-2-methylene-, (1S)- | β-pinene | C_10_ H_16_ | 136.1252 | 13.34 | 664033632 | Monoterpenes | 136.1246,  121.1011,  93.0698,  91.0542,  79.0542,  69.0698,  41.0385  27.0229 | [1] |
| 6 | β-Myrcene | β-Myrcene | C_10_ H_16_ | 136.1252 | 13.50 | 148922270 | Monoterpenes | 136.1246,  121.1011,  93.0698,  94.0733,  79.0542,  69.0698,  41.0385 | [1] |
| 7 | 2,3-Dehydro-1,8-cineole | dehydrocineole | C_10_ H_16_ O | 152.1201 | 13.60 | 31589043 | Monoterpenes | 152.119567, 124.0882664, 109.064791, 94.0777016, 79.054227, 69.033491 | [2] |
| 8 | α-Phellandrene | α-Phellandrene | C_10_ H_16_ | 136.1252 | 14.00 | 307279039 | Monoterpenes | 136.1246, 94.0733,  93.0698,  92.0620,  77.0385,  41.0385 | [3] |
| 9 | 3-Carene | 3-Carene | C_10_ H_16_ | 136.1252 | 14.16 | 35893294 | Monoterpenes | 136.1246,  121.1011,  94.0698,  92.0605,  79.0542,  77.0385,  43.0542,  41.0385 | [4] |
| 10 | 1,3-Cyclohexadiene, 1-methyl- 4-(1-methylethyl)- | α-Terpinene | C_10_ H_16_ | 136.1252 | 14.33 | 27996732 | Monoterpenes | 136.1246,  121.1011,  93.0698,  92.0620,  91.0542,  79.0542,  78.0464,  77.0385,  43.0542,  41.0385 | [1] |
| 11 | Benzene, 1-methyl-3-(1-methylethyl)- | m-cymene | C_10_ H_14_ | 134.1096 | 14.56 | 211403451 | Monoterpenes | 134.1090,  119.0855,  135.1124,  117.0698,  91.0542, 77.0385 | [3] |
| 12 | o-Cymene | o-Cymene | C_10_ H_14_ | 134.1096 | 14.67 | 556628815 | Monoterpenes | 134.1090,  119.0855,  117.0698,  91.0542,  135.1124,  89.0385,  77.0385 | [3] |
| 13 | Eucalyptol | Eucalyptol | C_10_ H_18_ O | 154.1358 | 14.83 | 1372391953 | Monoterpenes | 154.1352,  139.1117,  121.1011,  111.0804,  96.0933,  95.0855,  94.0777,  81.0698,  71.0491,  43.0178,  41.0385766 | [5] |
| 14 | 1,3,6-Octatriene, 3,7-dimethyl-, (Z)- | (Z)-ocimene | C_10_ H_16_ | 136.1252 | 15.05 | 34678619 | Monoterpenes | 136.1246,  121.1011,  105.0698,  95.0855,  94.0733,  93.0698,  92.0620,  80.0620,  79.0542,  53.0385,  63.0229,  41.0385 | [6] |
| 15 | γ-Terpinene | γ-Terpinene | C_10_ H_16_ | 136.1252 | 15.44 | 53431902 | Monoterpenes | 136.1246,  121.1011,  107.0855,  106.0777,  105.0698,  94.0733,  93.0698,  92.0620,  77.0385 | [1] |
| 16 | 5-Isopropyl-2-methylbicyclo[3.1.0]hexan-2-ol # | 4-thujanol | C_10_ H_18_ O | 154.1358 | 15.70 | 51102929 | Monoterpenes | 154.1352,  139.1117,  136.1246,  121.1011,  111.0804,  97.0647,  96.0569,  94.0777,  93.0698,  92.0620,  91.0542,  71.0491,  43.0542 | [6] |
| 17 | Cyclohexene, 3-methyl-6-(1-methylethylidene)- | Isoterpinolene | C_10_ H_16_ | 136.1252 | 16.25 | 60930038 | Monoterpenes | 136.1246,  121.1011,  107.0855,  95.0855,  94.0777,  93.0698,  92.0620,  91.0542,  79.0542,  67.0542,  41.0385 | [7] |
| 18 | Linalool | Linalool | C_10_ H_18_ O | 154.1358 | 16.45 | 164720609 | Monoterpenes | 154.1352,  136.1246,  121.1011,  93.0698,  80.0620,  71.0491, 69.0698,  55.0178,  43.0542,  41.0385 | [1] |
| 19 | Bicyclo[3.1.0]hexan-2-ol, 2-methyl-5-(1-methylethyl)-, (1α,2α,5α)- | 4-Thujanol | C_10_ H_18_ O | 154.1358 | 16.55 | 37420982 | Monoterpenes | 154.1352,  139.1117,  136.1246,  125.0960,  121.1011,  111.0804,  107.0855,  93.0698,  91.0542,  81.0698,  79.0542,  71.0491,  43.0542 | [7] |
| 20 | 2-Cyclohexen-1-ol, 1-methyl-4-(1-methylethyl)-, trans- | (E)-p-2-Menthen-1-ol | C_10_ H_18_ O | 154.1358 | 17.17 | 22314712 | Monoterpenes | 154.1352,  139.1117, 136.1246, 121.1011, 111.0804, 94.0777, 93.0698, 92.0620, 91.0542, 43.0542 | [8] |
| 21 | Bicyclo[3.1.1]heptan-3-ol, 6,6-dimethyl-2-methylene-, [1S-(1α,3α,5α)]- | laevo-pinocarveol | C_10_ H_16_ O | 152.1201 | 17.73 | 97763378 | Monoterpenes | 152.1195, 134.1090, 119.0855, 109.0647, 94.0777, 93.0698, 92.0620,91.0542, 83.0855, 70.0413, 69.0334, 55.0178, 41.0385 | [1] |
| 22 | trans-Verbenol | (E)-verbenol | C_10_H_16_O | 152.1201 | 17.81 | 35853884 | Monoterpenes | 152.1195, 137.0960, 119.0855, 110.0682, 109.0647, 108.0933, 107.0855, 94.0777, 91.0542,81.0698, 79.0542, 41.0385 | [1] |
| 23 | (+)-2-Bornanone | (+)-Camphor | C_10_ H_16_ O | 152.1201 | 17.89 | 239184686 | Monoterpenes | 152.1195, 137.0960, 109.1011, 108.0933, 95.0855, 81.0698, 69.0334, 55.0542, 41.0385 | [1] |
| 24 | Verbenol | Verbenol | C_10_ H_16_ O | 152.1201 | 18.15 | 18029976 | Monoterpenes | 152.1195, 137.0960, 119.0855, 109.0647, 94.0777, 91.0542, 79.0542, 41.0385 | [9] |
| 25 | Pinocarvone | Pinocarvone | C_10_ H_14_ O | 150.1045 | 18.31 | 22162195 | Monoterpenes | 150.1039, 135.0804, 108.0933, 81.0698, 79.0542, 53.0385, 54.0100, 41.0385 | [5] |
| 26 | Cyclohexanemethanol, α,α-dimethyl-4-methylene- | δ-Terpineol | C_10_ H_18_ O | 154.1358 | 18.37 | 111904796 | Monoterpenes | 140.1152, 139.1117, 136.1246, 96.0933, 93.0698, 81.0698, 69.0698, 60.0526, 59.0491, 43.0178 | [1] |
| 27 | Terpinen-4-ol | Terpinen-4-ol | C_10_ H_18_ O | 154.1358 | 18.66 | 172599758 | Monoterpenes | 154.1352, 136.1246, 111.0804, 93.0698,91.0542, 86.0726, 71.0491, 69.0698, 55.0542, 57.0698, 54.0464, 43.0542, 41.0385 | [10] |
| 28 | Benzenemethanol, α,α,4-trimethyl- | 8-Hydroxy-p-cymene | C_10_ H_14_ O | 150.1045 | 18.85 | 42033647 | Monoterpenes | 150.1039, 135.0804, 91.0542, 65.0385, 43.0178 | [3] |
| 29 | 2-Cyclohexen-1-one, 4-(1-methylethyl)- | 2-Cyclohexen-1-one, 4-(1-methylethyl)- | C_9_ H_14_ O | 138.1045 | 18.94 | 161651068 | Ketene | 138.1039, 96.0569, 95.0491, 81.0698, 67.0542, 65.0385, 43.0542, 41.0021 | [11] |
| 30 | α-Terpineol | α-Terpineol | C_10_ H_18_ O | 154.1358 | 19.04 | 650281154 | Monoterpenes | 140.1152, 136.1246, 121.1011, 93.0698, 92.0620, 81.0698, 67.0542, 59.0491, 43.0178 | [12] |
| 31 | Bicyclo[3.1.1]hept-2-ene-2-methan ol, 6,6-dimethyl- | (±)-Myrtenol | C_10_ H_16_ O | 152.1201 | 19.17 | 99493141 | Monoterpenes | 152.1195, 121.1011, 119.0855, 108.0933, 91.0542, 79.0542, 67.0542, 41.0385, 39.0229 | [1] |
| 32 | Bicyclo[3.1.1]hept-3-en-2-one, 4,6,6-trimethyl- | (S)-verbenone | C_10_ H_14_ O | 150.1045 | 19.50 | 20669355 | Ketones | 150.1039, 135.0804, 107.0855, 91.0542, 80.0620, 79.0542, 67.0542, 41.0385, 39.0229 | [13] |
| 33 | trans-Carveol | (E)-carveol | C_10_ H_16_ O | 152.1201 | 19.62 | 27770835 | Monoterpenes | 152.1195, 137.0960, 109.0647, 84.0569, 134.1090, 91.0542, 119.0855 | [14] |
| 34 | Benzaldehyde, 4-(1-methylethyl)- | 4-isopropylbenzaldehyde | C_10_ H_12_ O | 148.0888 | 20.21 | 35772061 | Monoterpenes | 148.0882, 133.0647, 119.0855, 105.0698, 91.0542, 77.0385 | [15] |
| 35 | (-)-Carvone | (-)-Carvone | C_10_H_14_O | 150.1045 | 20.27 | 34985133 | Monoterpenes | 150.1039, 108.0933, 107.0855, 93.0698, 82.0413, 55.0178, 54.0100 | [16] |
| 36 | 1-Cyclohexene-1-carboxaldehyde, 4-(1-methylethyl)- | Phellandral | C_10_ H_16_ O | 152.1201 | 21.10 | 37861224 | Monoterpenes | 152.1195, 137.0960, 109.0647, 95.0491, 81.0698, 79.0542, 67.0178, 41.0385 | [18] |
| 37 | (+)-Borneol acetate | Bornyl acetate | _C₁₂_H_₂₀O₂_ | 196.1463 | 21.24 | 28480033 | Monoterpenes | 196.1457, 154.1352, 136.1246, 121.1011, 96.0889, 95.0855, 94.0777, 93.0698 | [19] |
| 38 | p-Cymen-7-ol | Cuminol | C_10_H_14_O | 150.1045 | 21.34 | 42578334 | Aromatic alcohol | 150.1039, 135.0804, 119.0491, 107.0855, 105.0698, 91.0542, 79.0542 | [20] |
| 39 | Phenol, 2-methyl-5-(1-methylethyl)- | Phenol, 2-methyl-5-(1-methylethyl)- | C_10_ H_14_ O | 150.1045 | 21.51 | 55833594 | Monoterpenes | 150.1039, 136.0838, 135.0804, 117.0698, 107.0855, 91.0542, 77.0385 | [21] |
| 40 | n-Decanoic acid | Decanoic acid | C_₁₀H₂₀O₂_ | 172.1463 | 23.16 | 148691961 | Fatty acids | 172.14578, 143.1066, 129.0910, 115.0753, 87.0440, 84.0933, 43.0542,73.0284, 71.0855, 69.0698, 60.0205, 57.0334, 55.0542 | [22] |
| 41 | Copaene | Copaene | C_15_ H_24_ | 204.1878 | 23.46 | 241234238 | Sesquiterpenoids | 204.1872, 189.1637, 161.1324, 133.1011, 121.1011, 121.1011, 120.0933, 119.0855, 118.0777, 117.0698, 105.0698,91.0542 | [23] |
| 42 | (1R,2S,6S,7S,8S)-8-Isopropyl-1-methyl-3-methylenetricyclo[4.4.0.02,7]decane-rel- | β-copaene | C_15_ H_24_ | 204.1878 | 23.73 | 130096090 | Sesquiterpenoids | 204.1872, 161.1324, 147.1168, 133.1011, 120.0933, 118.0777, 119.0855, 105.0698, 91.0542, 55.0542, 41.0385 | [23] |
| 43 | Tricyclo[2.2.1.0(2,6)]heptane, 1,7-dimethyl-7-(4-methyl-3-pentenyl)-, (-)- | Santalene | C_15_ H_24_ | 204.1878 | 24.34 | 35979731 | Sesquiterpenoids | 204.1872, 189.1637, 161.1324, 133.1011, 121.1011, 107.0855, 105.0698, 94.0777, 79.0542, 69.0698, 41.0385 | [24] |
| 44 | Caryophyllene | Caryophyllene | C_15_ H_24_ | 204.1878 | 24.53 | 212393763 | Sesquiterpenoids | 204.1872, 189.1637, 175.1481, 161.1324, 147.1168, 133.1011, 120.0933, 105.0698, 93.0698,91.0542, 79.0542, 69.0698, 41.0385 | [25] |
| 45 | (1R,3aS,8aS)-7-Isopropyl-1,4-dimethyl-1,2,3,3a,6,8a-hexahydroazulene | (-)-Guaia-6,9-diene | C_15_ H_24_ | 204.1878 | 24.90 | 77362423 | Sesquiterpenoids | 204.1872, 189.1637, 161.1324, 147.1168, 133.1011, 119.0855, 105.0698, 91.0542, 41.0385 |  |
| 46 | Humulene | Humulene | C_15_H_24_ | 204.1878 | 25.26 | 143960828 | Sesquiterpenoids | 204.1872, 147.1168, 121.1011, 107.0855, 93.0698, 80.0620, 41.0385 | [3] |
| 47 | 2,5-Di-tert-butyl-1,4-benzoquinone | 2,5-Di-tert-butyl-1,4-benzoquinone | C_14_ H_20_ O_2_ | 220.1463 | 25.57 | 59319888 | Quinone | 220.1457, 205.1223, 177.1273, 163.0753, 149.0597, 135.0804, 121.0647, 91.0542, 41.0385 | [1] |
| 48 | Germacrene D | Germacrene D | C_15_ H_24_ | 204.1878 | 25.83 | 276793418 | Sesquiterpenoids | 204.1872, 161.1324, 133.1011, 119.0855, 105.0698, 91.0542, 81.0698, 77.0385, 41.0385 | [2] |
| 49 | Naphthalene, decahy dro-4a-methyl-1-methylene-7-(1-methylethenyl)-, [4aR-(4aα,7α,8aβ)]- | β-selinene | C_15_ H_24_ | 204.1878 | 25.97 | 79991891 | Sesquiterpenoids | 204.1872, 189.1637, 175.1481, 161.1324, 147.1168, 133.1011, 121.1011, 107.0855, 105.0698,93.0698, 79.0542, 67.0542 | [3] |
| 50 | (1S,2E,6E,10R)-3,7,11,11-Tetramethylbicyclo[8.1.0]undeca-2,6-diene | bicyclogermacrene | C_15_ H_24_ | 204.1878 | 26.16 | 270564334 | Sesquiterpenoids | 204.1872, 189.1637, 161.1324, 147.1168, 121.1011, 93.0698, 91.0542, 79.0542, 41.0385 | [1] |
| 51 | Azulene, 1,2,3,5,6,7,8,8a-octahydro-1,4-dimethyl-7-(1-methylethenyl)-, [1S-(1α,7α,8aβ)]- | α-Bulnesene | C_15_ H_24_ | 204.1878 | 26.25 | 21423456 | Sesquiterpenoids | 204.1872, 189.1637, 147.1168, 135.1168, 121.1011, 108.0933, 55.0542, 41.0385 | [26] |
| 52 | 1-Isopropyl-4,7-dimeth yl-1,2,3,5,6,8a-hexahydronaphthalene | α-himachalene | C_15_ H_24_ | 204.1878 | 26.59 | 68750670 | Sesquiterpenoids | 204.1872, 189.1637, 161.1324, 134.1090, 119.0855, 105.0698, 91.0542, 69.0698, 41.0385 | [27] |
| 53 | Kessane | Kessane | C_15_ H_26_ O | 222.1984 | 27.01 | 60311509 | Sesquiterpenoids | 222.1978, 189.1637, 149.1324, 126.1039, 109.0647, 81.0698, 55.0542, 43.0178 | [28] |
| 54 | Cyclohexanemethanol, 4-ethenyl-α,α,4-trimethyl-3-(1-methylethenyl)-, [1R-(1α,3α,4β)]- | α-elemol | C_15_ H_26_ O | 222.1984 | 27.17 | 531208229 | Sesquiterpenoids | 207.1743, 204.1872, 161.1324, 107.0855, 93.0698, 81.0698, 59.0491, 41.0385, 43.0178 | [1] |
| 55 | 2H-3,9a-Methano-1-benzoxepin, octahydro-2,2,5a,9-tetramethyl-, [3R-(3α,5aα ,9α,9aα)]- | α-himachalene | C_15_ H_26_ O | 222.1984 | 27.51 | 96059469 | Sesquiterpenoids | 222.1978, 207.1743, 189.1637, 149.1324, 137.1324, 123.1168, 109.1011, 107.0855, 81.0698, 55.0542, 41.0385 | [28] |
| 56 | 1H-Cycloprop[e]azulen-7-ol, decahydro-1,1,7-trimethyl-4-methylene-, [1ar-(1aα,4aα,7β,7aβ,7bα)]- | Spathulenol | C_15_ H_24_ O | 220.1827 | 27.88 | 415060215 | Sesquiterpenoids | 220.1821, 205.1586, 187.1481, 159.1168, 147.1168,131.0855, 119.0855, 122.1090, 121.1011, 120.0933, 118.0777, 117.0698, 91.0542, 43.0178 | [29] |
| 57 | Caryophyllene oxide | Caryophyllene oxide | C₁₅H₂₄O | 220.1827 | 28.01 | 217782467 | Sesquiterpenoids | 220.1821, 121.1011, 93.0698, 91.0542, 79.0542, 81.0698, 80.0620, 43.0178, 41.0385 | [30] |
| 58 | Guaiol | Guaiol | C_15_ H_26_ O | 222.1984 | 28.14 | 185018381 | Sesquiterpenoids | 222.1978, 204.1872, 189.1637, 163.1481, 162.1403, 161.1324, 160.1246, 107.0855, 59.0491, 41.0385 | [1] |
| 59 | 2-Naphthalenemethanol, 2,3,4,4a,5,6,7,8-octahydro-α,α,4a,8-tetramethyl-, [2R-(2α,4aβ,8β)]- | Rosifoliol | C_15_ H_26_ O | 222.1984 | 28.33 | 381023200 | Sesquiterpenoids | 205.1907, 164.1559, 149.1324, 135.1168, 121.1011, 150.1359, 147.1168, 108.0933, 59.0491 | [31] |
| 60 | 2-Naphthalenemethanol, 1,2,3,4,4a,5,6,7-octahydro-α,α,4a,8-tetramethyl-, (2R-cis)- | gamma-eudesmol | C_15_ H_26_ O | 222.1984 | 28.49 | 481529380 | Sesquiterpenoids | 222.1978, 204.1872, 189.1637, 161.1324, 164.1515, 162.1403, 133.1011, 107.0855, 93.0698 | [32] |
| 61 | Cubenol | Cubenol | C_15_ H_26_ O | 222.1984 | 28.70 | 39693474 | Sesquiterpenoids | 207.1743, 204.1872, 161.1324, 119.0855, 105.0698, 93.0334, 81.0698, 79.0542, 43.0542, 41.0385 | [33] |
| 62 | 2-Naphthalenemethanol, 1,2,3,4, 4a,5,6,7-octahydro-α,α,4a,8-tetramethyl-, (2R-cis)- | 2-Naphthalenemethanol, 1,2,3,4,4 a,5,6,*7-octahydro-α*,*α*,4a,8-tetramethyl-, (2R-cis)- | C_15_H_26_O | 222.1984 | 28.80 | 179383982 | Sesquiterpenoids | 222.1978, 204.1872, 189.1637, 161.1324, 149.1324, 133.1011, 93.0698, 59.0491 | [32] |
| 63 | 2-Naphthalenemethanol, 1,2,3,4,4a,5,6,8a-octahydro-α,α,4a,8-tetramethyl-, [2R-(2α,4aα,8aβ)]- | α-eudesmol | C_15_H_26_O | 222.1984 | 29.20 | 596729215 | Sesquiterpenoids |  | [34] |
| 64 | 5-Azulenemethanol, 1,2,3,3a,4,5,6,7-octahydro-α,α,3,8-tetramethyl-, [3S-(3α,3aβ,5α)]- | 5-Azulenemethanol, 1,2,3,3a,4,5,6,*7-octahydro-α*,*α*,3,8-tetramethyl-, [3S-(*3α*,*3aβ*,*5α*)]- | C_15_ H_26_ O | 222.1984 | 29.39 | 125128221 | Sesquiterpenoids | 222.1978, 189.1637, 161.1324, 135.1168, 108.0933, 107.0855, 105.0698, 106.0777 | [1] |
| 65 | Guaiol | Guaiol | C_15_ H_26_ O | 222.1984 | 29.65 | 21245063 | Sesquiterpenoids |  | [1] |
| 66 | Dodecanoic acid, 2-[(1-oxodecyl)oxy]-1-[[(1-oxodecyl)oxy]methyl]ethyl ester | Dodecanoic acid, 2-[(1-oxodecyl)oxy]-1-[[(1-oxodecyl)oxy]methyl]ethyl ester | C_35_ H_66_ O_6_ | 582.4859 | 38.39 | 661248422 | Fatty acid esters |  |  |
| 67 | Laurin, 2-capri-1,3-di- | Laurin, 2-capri-1,3-di- | C_37_ H_70_ O_6_ | 610.5172 | 48.60 | 322815250 | Fatty acid esters |  | [35] |
| 68 | Decanoic acid, 1,2,3-propanetriyl ester | Decanoic acid, 1,2,3-propanetriyl ester | C_33_ H_62_ O_6_ | 554.4546 | 53.61 | 116959666 | Fatty acid esters |  | [36] |

**References**

[1] Wang X.J. Study on Serum Medicinal Chemistry of Traditional Chinese Medicine and Chinese Medicine Compounds. World Sci. Technol. 2002;4:1–4,+78.
[2] Puzhu Cong, Sunyu Li. Natural Organic Mass Spectrometry. Beijing: China Medical Science Press; 2003.
[3] Paste volatiles[J]. Journal of Agricultural and Food Chemistry, 1990,38(1):336-340.
[4] Yuehua Wu**.** *Application of thermal desorption for aroma component analysis of rose and frankincense* [Doctoral Thesis]. Lanzhou: Gansu Agricultural University; 2013.
[5] Qunying Gao, Yan Gao, Ruming Zhang, et al. Analysis of aroma components in three Asteraceae plants by thermal desorption coupled with gas chromatography-mass spectrometry. Journal of Zhejiang A&F University 2011; 28(2): 326–332.
[6] MeiLing Cheng, Yan Sun, Rui Liu, et al. Analysis of volatile oil components from *Angelica dahurica* extracted by different methods using GC-MS combined with retention indices. *Chinese Journal of Pharmaceutical Analysis* 2024; 44(6): 921-928. DOI: 10.16155/j.0254-1793.2024.06.01.
[7] de Almeida Teles AC, Dos Santos BO, Santana EC, Durço AO, Conceição LSR, Roman-Campos D, de Holanda Cavalcanti SC, de Souza Araujo AA, Dos Santos MRV. Larvicidal activity of terpenes and their derivatives against Aedes aegypti: a systematic review and meta-analysis. Environ Sci Pollut Res Int. 2024 Dec;31(56):64703-64718. doi: 10.1007/s11356-024-35479-w. Epub 2024 Nov 16. PMID: 39549195.
[8] Ana AG, Carrillo-Cerda HA, Rodriguez-Campos J, Velázquez-Fernández JB, Patrón-Soberano OA, Contreras-Ramos SM. Dynamics of volatilomes emitted during cross-talking of plant-growth-promoting bacteria and the phytopathogen, Fusarium solani. World J Microbiol Biotechnol. 2020 Sep 14;36(10):152. doi: 10.1007/s11274-020-02928-w. PMID: 32924087.
[9] Röpke R, Millar JG, Halloran S, Ginzel MD, Tobin KN, Grommes AC, Hanks LM. (1R,4R)-Quercivorol, a Male-Produced Aggregation-Sex Pheromone of the Beetle Elytrimitatrix undata (F.) (Coleoptera: Disteniidae). Journal of Chemical Ecology. 2023 Aug;49(7-8):363-368. doi: 10.1007/s10886-023-01426-1. Epub 2023 Apr 21. PMID: 37085723.
[10] Hhuajie Zhao, Ning Guo, Lingxiao Yang, et al. Extraction and analysis of volatile components from dried fruits of *Litsea pungens*. *Flavour Fragrance Cosmetics* 2017; (5): 1-5.
[11] Dandan Pu, Yimeng Shan, Yuyu Zhang. Research progress on aroma components, bioactivities and metabolic pathways of *Litsea pungens*. *Science and Technology of Food Industry* 2022; 43(17): 435-448.
[12] de Oliveira FS, Azambuja M, Schemberger MO, Nascimento VD, Oliveira JIN, Wolf IR, Nogaroto V, Martins C, Vicari MR. Characterisation of the hAT DNA transposon superfamily in the genome of the Neotropical fish Apareiodon sp. Molecular Genetics and Genomics. 9 October 2024;299(1):96. doi: 10.1007/s00438-024-02190-x. PMID: 39382723.
[13] Na Fan. *Study on the pharmacodynamic substance basis and mechanism of Platycladi Cacumen in promoting hair growth* [Doctoral dissertation]. Xi'an: No rthwest University; 2022.
[14] Yuvaraj JK, Kandasamy D, Roberts RE, Hansson BS, Gershenzon J, Andersson MN. Eurasian spruce bark beetle detects lanierone using a highly expressed specialist odorant receptor, present in several functional sensillum types. BMC Biol. 2024 Nov 20;22(1):266. doi: 10.1186/s12915-024-02066-x. PMID: 39568015; PMCID: PMC11577813.
[15] Ma M, Fu H, Wang T, Xiong L, Feng P, Lu B. Widely targeted volatilomics and transcriptome analyses reveal the differences in volatile organic components in differently shaped Amomum tsao-ko fruits. BMC Plant Biol. 1 October 2024;24(1):915. doi: 10.1186/s12870-024-05594-4. PMID: 39350013; PMCID: PMC11443856.
[16] Xue CB, Zhang L, Luo WC, Xie XY, Jiang L, Xiao T. 3D-QSAR and molecular docking studies of benzaldehyde thiosemicarbazone, benzaldehyde, benzoic acid, and their derivatives as phenoloxidase inhibitors. Bioorganic & Medicinal Chemistry. 1 March 2007;15(5):2006-15. doi: 10.1016/j.bmc.2006.12.038. Epub 2006 Dec 30. PMID: 17258462.
[17] de Sousa DP, Mesquita RF, de Araújo Ribeiro LA, de Lima JT. Spasmolytic Activity of Carvone and Limonene Enantiomers. Nat Prod Commun. 2015 Nov;10(11):1893-6. PMID: 26749818.
[18] Pandey VK, Tripathi A, Srivastava S, Dar AH, Singh R, Farooqui A, Pandey S. Exploiting the bioactive properties of essential oils and their potential applications in the food industry. Food Sci Biotechnol. 24 March 2023;32(7):885-902. doi: 10.1007/s10068-023-01287-0. PMID: 37123062; PMCID: PMC10130317.
[19] Sgorbini B, Bicchi C, Cagliero C, Cordero C, Liberto E, Rubiolo P. Herbs and spices: characterisation and quantitation of biologically-active markers for routine quality control by multiple headspace solid-phase microextraction combined with separative or non-separative analysis. J Chromatogr A. 9 January 2015;1376:9-17. doi: 10.1016/j.chroma.2014.12.007. Epub 10 December 2014. PMID: 25541091.
[20] Strijkstra A, Trautwein K, Jarling R, Wöhlbrand L, Dörries M, Reinhardt R, Drozdowska M, Golding BT, Wilkes H, Rabus R. Anaerobic activation of p-cymene in denitrifying β-proteobacteria: methyl group hydroxylation versus addition to fumarate. Appl Environ Microbiol. 2014 Dec;80(24):7592-603. doi: 10.1128/AEM.02385-14. Epub 2014 Sep 26. PMID: 25261521; PMCID: PMC4249252.
[21] Feipeng Gong. Study on chemical constituents and pharmacodynamic effects of Atractylodes lancea processed with rice-washed water [Doctoral dissertation]. Nanchang: Jiangxi University of Chinese Medicine; 2024.
[22] Rollero S, Bloem A, Camarasa C, Sanchez I, Ortiz-Julien A, Sablayrolles JM, Dequin S, Mouret JR. Combined effects of nutrients and temperature on the production of fermentative aromas by Saccharomyces cerevisiae during wine fermentation. Applied and Environmental Microbiology. 2015 Mar;99(5):2291-304. doi: 10.1007/s00253-014-6210-9. Epub 2014 Nov 21. PMID: 25412578.
[23] Hhuajun Wang, Xxiaofu Li, Wenjie Zhang, et al. Analysis of volatile aroma components in Zanthoxylum bungeanum extracted by different methods. China Condiment 2020; 45(6): 49–53.
[24] Chen X, Zhang Y, Yan H, Niu M, Xiong Y, Zhang X, Li Y, Teixeira d a Silva JA, Ma G. Cloning and functional analysis of 1-deoxy-d-xylulose-5-phosphate synthase (DXS) in Santalum album L. Gene. 2023 Jan 30;851:146762. doi: 10.1016/j.gene.2022.146762. Epub 2022 Aug 3. Erratum in: Gene. 2024 Dec 30;931:148878. doi: 10.1016/j.gene.2024.148878. PMID: 35933050.
[25] Jieyi Liang, Yangchun Xu, Weijia Li, et al. Optimisation of extraction technology and GC-MS identification of volatile oil from Rhodomyrtus tomentosa fruits. Food Industry 2018; 39(12): 124–127.
[26] Hui Ran, Lulu Feng, Yunzhi Mao, et al. GC-MS identification and volatile component analysis of leaf essential oils from four wild Lauraceae species in Chongqing. *Scientia Silvae Sinicae* 2018; 54(7): 91-103.
[27] Yukun Wei, Haocheng Wei, Ling Wu, et al. Analysis of volatile compounds in cultured large yellow croaker from Ningde by SPME/SDE-GC-MS. Food Research and Development 2020; 41(15): 129–136.
[28] Verma RS, Verma RK, Padalia RC, Chauhan A, Singh A, Singh HP. Chemical diversity in the essential oil of Indian valerian (Valeriana jatamansi Jones). Chem Biodivers. 2011 Oct;8(10):1921-9. doi: 10.1002/cbdv.201100059. PMID: 22006721.
[29] Reilly CB, Ramarathinam SH, Williamson NA, Purcell AW. Stable isotope shifted matrices enable the use of low mass ion precursor scanning for targeted metabolite identification. Proteome Sci. 2011 Jan 17;9(1):2. doi: 10.1186/1477-5956-9-2. PMID: 21241510; PMCID: PMC3025931.
[30] Muñoz-Acevedo A, González MC, Alonso JE, Flórez KC. The Repellent Capacity against Sitophilus zeamais (Coleoptera: Curculionidae) and In Vitro Inhibition of the Acetylcholinesterase Enzyme of 11 Essential Oils from Six Plants of the Caribbean Region of Colombia. Molecules. 2024 Apr 12;29(8):1753. doi: 10.3390/molecules29081753. PMID: 38675573; PMCID: PMC11051817.
[31] Gachet MS, Kunert O, Kaiser M, Brun R, Zehl M, Keller W, Muñoz RA, Bauer R, Schuehly W. Antiparasitic compounds from Cupania cinerea with activities against Plasmodium falciparum and Trypanosoma brucei rhodesiense. Journal of Natural Products. 25 April 2011;74(4):559-66. doi: 10.1021/np100415m. Epub 2011 Mar 25. PMID: 21438586.
[32] Peng Y, Bishop KS, Quek SY. Compositional Analysis and Aroma Evaluation of Feijoa Essential Oils from New Zealand Grown Cultivars. Molecules. 2019 May 29;24(11):2053. doi: 10.3390/molecules24112053. PMID: 31146480; PMCID: PMC6600377.
[33] Janta P, Vimolmangkang S. Chemical profiling and clustering of various dried cannabis flowers revealed by volatilomics and chemometric processing. J Cannabis Res. 6 December 2024;6(1):41. doi: 10.1186/s42238-024-00252-w. PMID: 39639406; PMCID: PMC11622677.
[34] Dickschat JS, Quan Z, Schnakenburg G. A Case of Convergent Evolution: The Bacterial Sesquiterpene Synthase for 1-epi-Cubenol from Nonomuraea coxensis. Chembiochem. 2023 Dec 1;24(23):e202300581.
[35] Costa EV, Alencar Menezes LR, Dutra LM, Pinheiro MLB, Lavor ÉM, Silva MG, Alves CDSC, Almeida JRGS, da Silva FMA, Koolen HHF, Barison A. A novel eudesmol derivative from the leaf essential oil of Guatteria friesiana (Annonaceae) and evaluation of the antinociceptive activity. Z Naturforsch C J Biosci. 18 May 2022;78(5-6):169-177. doi: 10.1515/z nc-2022-0059. PMID: 35579663.
[36] Lingwood D, Simons K. Lipid rafts as a membrane-organising principle. Science. 1 January 2010;327(5961):46–50.
