# Supplementary material for: Integrating multi-omics and deep learning to explore the active ingredients and molecular mechanisms of Cinnamomum migao volatile oil in the treatment of coronary heart disease
Source: Front Pharmacol. 2026 Aug 5;17:1823482. doi: 10.3389/fphar.2026.1823482 (PMC13486190; doi:10.3389/fphar.2026.1823482)
Supplement: Supplementary file 4 [file Supplementaryfile2.zip › Supplementary Data S2 Raw GC–MS data for serum pharmacochemistry analysis/Blank_Serum_TIC.pdf.pdf]

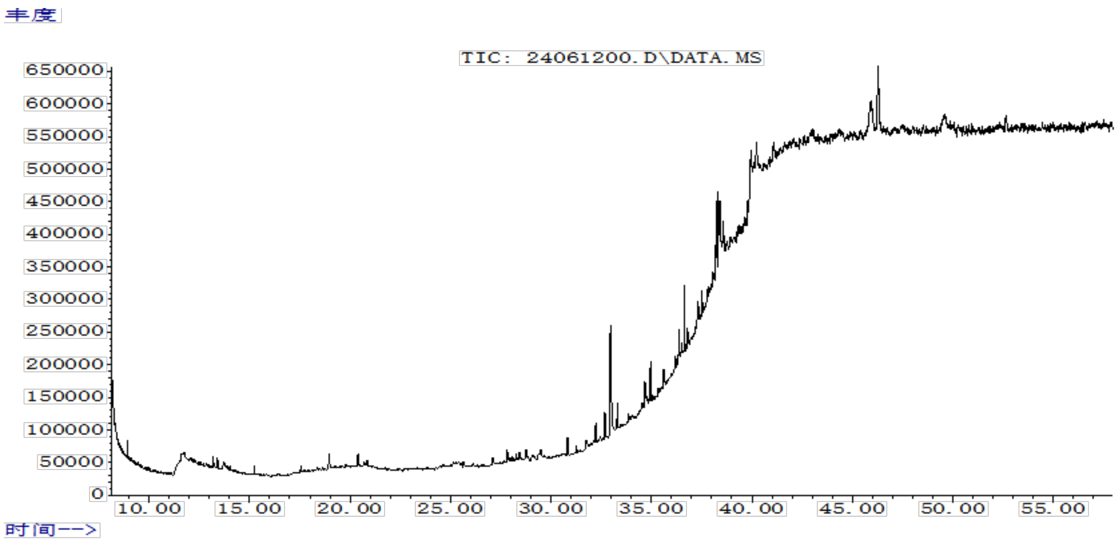
丰度
TIC: 24061200.D\DATA.MS
650000
600000
550000
500000
450000
400000
350000
300000
250000
200000
150000
100000
50000
0
10.00
15.00
20.00
25.00
30.00
35.00
40.00
45.00
50.00
55.00
时间-->
