# Supplementary material for: Integrating multi-omics and deep learning to explore the active ingredients and molecular mechanisms of Cinnamomum migao volatile oil in the treatment of coronary heart disease
Source: Front Pharmacol. 2026 Aug 5;17:1823482. doi: 10.3389/fphar.2026.1823482 (PMC13486190; doi:10.3389/fphar.2026.1823482)
Supplement: Supplementary file 4 [file Supplementaryfile2.zip › Supplementary Data S2 Raw GC–MS data for serum pharmacochemistry analysis/CMVO_Containing_Serum_TIC.pdf.pdf]

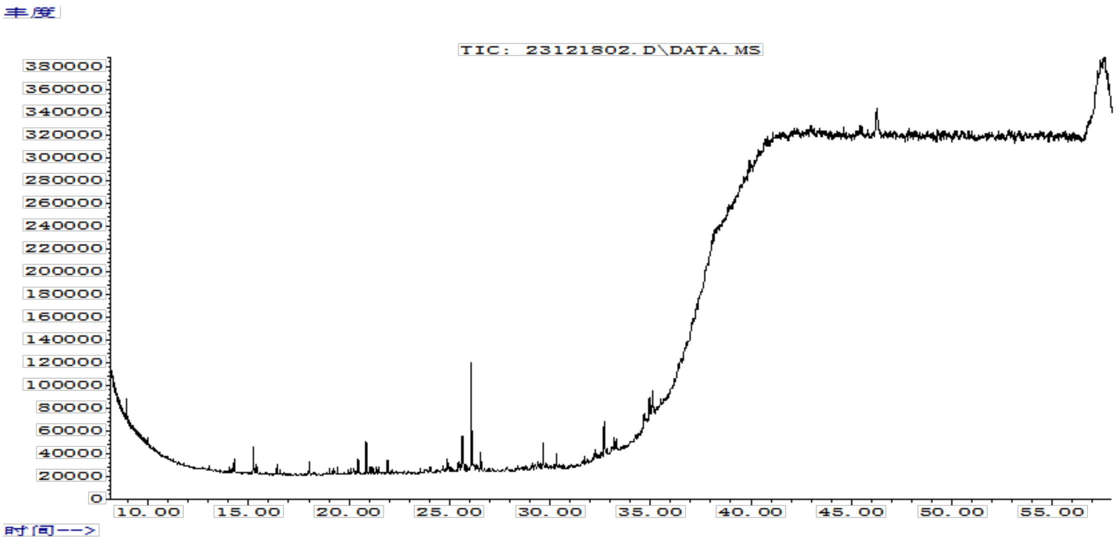
丰度
TIC: 23121802.D\DATA.MS
380000
360000
340000
320000
300000
280000
260000
240000
220000
200000
180000
160000
140000
120000
100000
80000
60000
40000
20000
0
10.00
15.00
20.00
25.00
30.00
35.00
40.00
45.00
50.00
55.00
时间-->
