# Supplementary material for: Integrating multi-omics and deep learning to explore the active ingredients and molecular mechanisms of Cinnamomum migao volatile oil in the treatment of coronary heart disease
Source: Front Pharmacol. 2026 Aug 5;17:1823482. doi: 10.3389/fphar.2026.1823482 (PMC13486190; doi:10.3389/fphar.2026.1823482)
Supplement: Supplementary file 5 [file Supplementaryfile3.zip › Supplementary Data S1 Raw GC–MS data for the chemical profiling of CM-VO/CMVO_TIC.pdf.pdf]

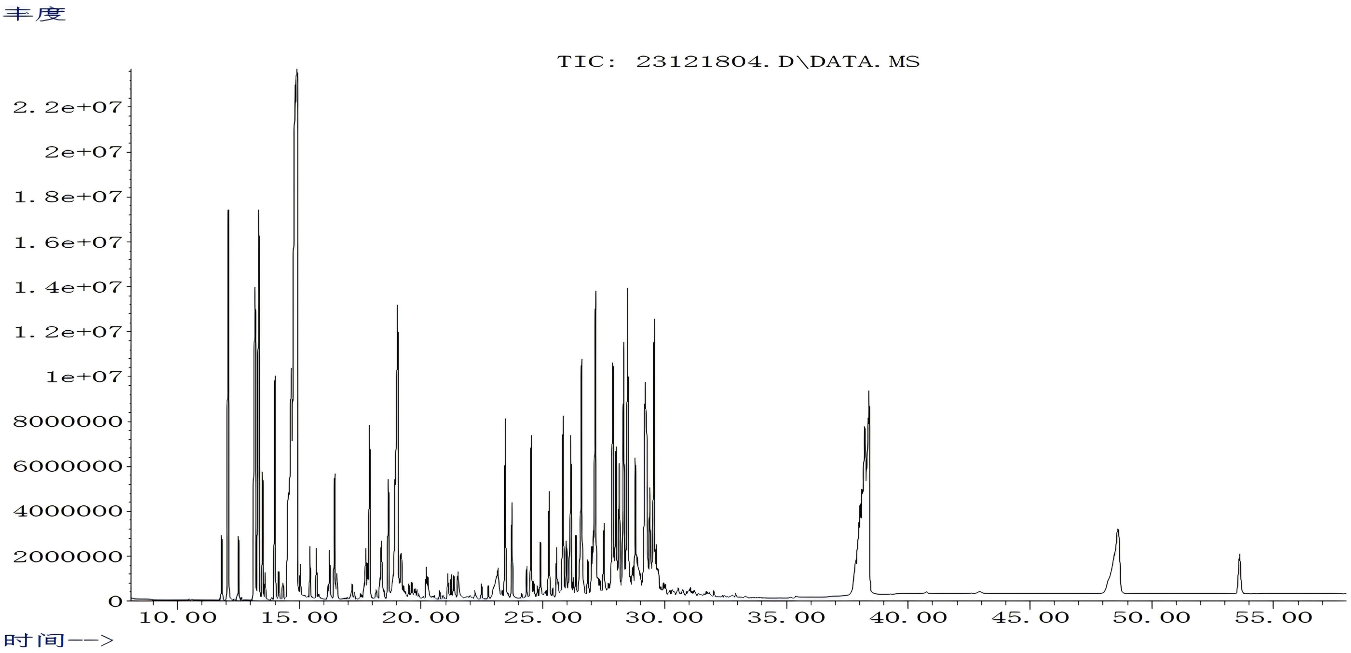
丰度
TIC: 23121804.D\DATA.MS
2.2e+07
2e+07
1.8e+07
1.6e+07
1.4e+07
1.2e+07
1e+07
8000000
6000000
4000000
2000000
0
10.00
15.00
20.00
25.00
30.00
35.00
40.00
45.00
50.00
55.00
时间-->
